# Potential of *Aeginetia indica* for Alzheimer's Disease Management: In Vitro, In Vivo, and Computational Insights

**DOI:** 10.1002/fsn3.71886

**Published:** 2026-06-19

**Authors:** Marjanur Rahman Bhuiyan, Md. Sharif Reza, Sadikur Rahman Shuvo, Mustafa Abdullah Yilmaz, Syed Mumtahin Mannan Siam, Md. Shadman Sakib, Oguz Cakir, Md. Nazmul Islam Durjoy, Abbas Tarhan, Nuraniye Eruygur, A. F. M. Shahid Ud Daula

**Affiliations:** ^1^ Department of Pharmacy, Faculty of Science Noakhali Science and Technology University Noakhali Bangladesh; ^2^ Department of Microbiology Noakhali Science and Technology University Sonapur Noakhali Bangladesh; ^3^ Dicle University Science and Technology Research and Application Center Diyarbakir Turkey; ^4^ Department of Analytical Chemistry, Faculty of Pharmacy Dicle University Diyarbakir Turkey; ^5^ Department of Nutrition and Dietetics, Faculty of Health Sciences Dicle University Diyarbakir Turkey; ^6^ Department of Pharmacognosy, Faculty of Pharmacy Selcuk University Konya Turkey

**Keywords:** carrageenan induced anti‐inflammatory assay, DPPH‐free radical scavenging assay, ferric‐reducing antioxidant power assay, LCMS/MS and GCMS analysis, molecular docking and dynamics simulation, reducing power activity assay

## Abstract

The 2023 Alzheimer's Disease International report warns that Alzheimer's disease (AD) is an escalating global challenge, with estimates suggesting more than 150 million cases worldwide by 2050 and about 13.8 million in the United States by 2060. AD is often linked with cholinergic dysfunction and increased acetylcholinesterase (AChE) activity, exacerbated by chronic neuroinflammation and oxidative stress. This study investigates the potential of *Aeginetia indica* whole plant methanolic extract (AiME) and its phytochemicals as natural AChE inhibitors (AChEIs) for AD management. AiME (IC_50_ = 176.586 ± 5.96 μg/mL) exhibited moderate, dose‐dependent antioxidant activity compared to standard ascorbic acid (IC_50_ = 38.866 ± 10.059 μg/mL) in the DPPH antioxidant assay. The other two antioxidant assays, reducing power activity (RPA) and ferric‐reducing antioxidant power (FRAP), also followed a similar trend (AiME: EC_50_ = 358.249 ± 16.605 μg/mL, EC_50_ = 39.467 ± 9.046 μg/mL), respectively, compared to standard ascorbic acid (Std: EC_50_ = 53.778 ± 0.624 μg/mL, Std: EC_50_ = 48.986 ± 0.512 μg/mL). AiME at all doses showed significant inhibition (*p* < 0.001) of paw edema in animals (in vivo) compared to the negative control group. Notably, AiME displayed better AChE inhibitory activity (IC_50_ = 1.636 ± 0.067 mg/mL) compared to standard rivastigmine (IC_50_ = 2.344 ± 0.151 mg/mL) in the acetylcholinesterase assay. LCMS/MS analysis identified 16 phenolic compounds, and GCMS analysis revealed 23 phytoconstituents in AiME. Molecular docking studies revealed acteoside, acacetin, and luteolin as promising leads, exhibiting favorable binding affinities and hydrogen bond interactions with the AChE active site. The structural stability and rigidity of our top two lead compounds, acteoside and acacetin, were evaluated using 100 ns molecular dynamics simulations, where they exhibited lower root mean square deviation values, with the acteoside‐AChE complex ranging from 0.1 to 0.35 nm and the acacetin‐AChE complex ranging from 0.1 to 0.4 nm, indicating notable stability. This study highlights the potential of *A. indica* and its phenolic constituents as natural alternatives for AD management, offering a multi‐pronged mechanistic pathway through AChE inhibition, anti‐inflammatory, and antioxidant properties.

## Graphical abstract

MeOH extract of *Aeginetia indica* (AİME); chromatographic study: LCMS/MS and GCMS; in vitro assays: AChE assays → ↓ AChE level, ↓ inflammation level and antioxidant assays → ROS; in vivo anti‐inflammatory study: ↓ inflammation level; molecular docking → molecular dynamics simulations: acacetin and acteoside.

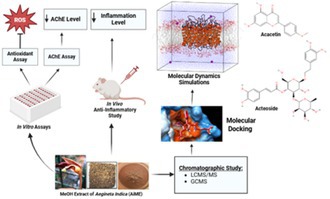

## 1 Introduction

Alzheimer's disease (AD) is one of the most prevalent neurodegenerative disorders, characterized by a profound decline in cognitive functions (Yang et al. 2017). It has become a significant concern among older patients affecting around 5.5 million people in the US alone (Rehman et al. 2022). The Alzheimer's Disease International 2023 report (“2023 Alzheimer's Disease Facts and Figures” 2023) has projected that the total number of AD cases will increase from 50 million in 2018 to approximately 150 million by 2050, with about 13.8 million in the United States by 2060, and it is also expected to witness a doubling in the number of cases in Europe (Li et al. 2022; Scheltens et al. 2021). During the early stages of AD, patients experience mild symptoms such as memory loss, behavioral changes, and impaired communication skills. As the disease progresses, these symptoms lead to a severe decline in cognitive functions including movement disabilities, complete memory loss, and disorientation (Rehman et al. 2022).

Two hallmark pathological features of Alzheimer's disease are neurofibrillary tangles (NFTs) and senile plaques (SPs) (Kumar et al. 2015; Scheltens et al. 2021; Yang et al. 2017). Senile plaques are amyloid plaques that contribute to cerebral amyloid angiopathy (CAA) through the deposition of Aβ proteins in the brain's parenchyma and cerebral blood vessels (Kumar et al. 2015). Aβ42 isoforms of amyloid‐β (Aβ) are particularly critical for the progression of AD, as they exhibit greater aggregation propensity and neurotoxicity compared to other isoforms, Aβ40 (Yang et al. 2017). On the other hand, NFTs arise from the hyperphosphorylation of tau protein within neurons, following the steps of neuroinflammation in Alzheimer's disease patients' brains (Jain et al. 2024). Consequently, the acetylcholinesterase enzyme (AChE) becomes upregulated, leading to the hydrolysis of acetylcholine (Ach), a vital neurotransmitter at the synaptic cleft. This process causes a deficiency of acetylcholine at nerve endings, impairing nerve impulses and contributing significantly to the progression of AD.

Acetylcholinesterase inhibitors (AChEIs) are the first line therapeutic agents currently considered for patients with Alzheimer's disease. AChEIs restore diminished acetylcholine (Ach) levels at the synaptic cleft by blocking the upregulation of AChE, a direct consequence of hyperphosphorylation of tau proteins (NFTs), one hallmark of AD described above. Several AChEIs, such as rivastigmine, donepezil, and galantamine, have received approval for Alzheimer's disease treatment from regulatory bodies in more than 60 countries (Birks and Grimley Evans 2015). However, the efficacy of these drugs remains a subject of debate, with some studies claiming half of the populations treated with these drugs ended up with no efficient response (Kumar et al. 2015). Additionally, AChEIs are also reported with potential serious cholinergic adverse effects including hepatotoxicity, bone fractures, eczema rash, unusual weakness, gastrointestinal disturbances, and nocturia (Rehman et al. 2022). These harmful side effects limit their clinical use in patients and demand for developing natural sources‐based alternatives with a more favorable safety profile.

Moreover, more recent research suggests persistent neuroinflammation and oxidative stress in the brain as another third core pathophysiological mechanism in AD, alongside the two established hallmarks of SPs and NFTs (Agostinho et al. 2010). Therefore, considering the limitations of current therapeutic options and the recently proposed link between neuroinflammation and oxidative stress in Alzheimer's disease, we aim to investigate the antioxidant, anti‐inflammatory, and acetylcholinesterase (AChE) inhibitory activities of the parasitic plant *Aeginetia indica* , which has a history of medicinal use in Asia (On‐Nom et al. 2024).

The parasitic herb plant *A. indica* predominantly grows in various Asian countries (Liu et al. 2012). It is especially abundant in the Chittagong Hill Tracts of Bangladesh. This plant is well known by the two local names “Min Mukhi” and “Buishakful Gulu” inside Bangladesh. *Aeginetia indica* has a rich history for consuming in various ways across Asia. For instance, Thai people use it as a food colorant in their different desserts (Liu et al. 2012). In Nepal, the root juice from this herb is used to treat fever (Sapkota 2014). In Myanmar and New Guinea, the whole plant of *A. indica* was consumed orally for knee pain, throat discomfort, and urinary tract infections (Hong et al. 2015; Parnell 2001). Several other studies also reported the various uses of *A. indica* for different pharmacological activities against Hepatitis C and renal cancer cell growth (Lin et al. 2018), angiogenesis (Bhuiyan et al. 2025). Few other pharmacological activities from *A. indica* , such as analgesic, antipyretic, antidiabetic, and hepatoprotective activities, were also reported from our lab (Reza et al. 2021, 2020). The diverse traditional medicinal uses of *A. indica* in Asia, together with previous investigations from our laboratory demonstrating its antioxidant and anti‐inflammatory effects, led us to extend the current study to evaluate acetylcholinesterase inhibitory activity (AChEI). Our hypothesis focused on investigating neurological pathways such as neuroinflammation and oxidative stress associated with AD development by targeting these pharmacological activities. Thus, we aimed to evaluate, for the first time, the potential of *A. indica* in blocking the progression of Alzheimer's disease by simultaneously targeting three key mechanisms: senile plaques (SPs), neurofibrillary tangles (NFTs), and neuroinflammation/oxidative stress. In addition, we sought to explore other phytoactive compounds from *A. indica* , including acteoside, which has previously been reported to contribute to the disposition of senile plaques through a multifaceted approach (Kurisu et al. 2013; Shiao et al. 2017).

## 2 Materials & Methodology

### 2.1 Plant Extract Preparation

*Aeginetia indica* whole plant picked up from Chandranath Hill, Chittagong, Bangladesh (22°50′59″ N 91°38′14″ E) during winter season in January 2020. Khandakar Kamrul Islam, an expert Scientific Officer of the Bangladesh National Herbarium, verified its authenticity of the plant with previously voucher specimen (DACB No‐46478). The collected plants were cleaned from the dirt properly and left in the air dry for 7 days. Then the collected plant was washed thoroughly to remove all kinds of dirt and left to air dry for 7 days before crushing into fine powder. A total of 130.53 g of fine powder was then soaked in 80% methanol for 14 days. To obtain a clear filtrate from the suspension, we used a cloth filter first and then a Whatman filter paper. The final crude methanolic extract of *A. indica* (AiME, 7.5%) was prepared by concentrating the clear filtrate via rotary evaporator under reduced pressure at 40°C for 6 h. A total of 9.6 g of yellowish‐brown extract was then stored at 4°C until further use.

### 2.2 In Vitro Antioxidant Assay

#### 2.2.1 DPPH‐Free Radical Scavenging Assay

The DPPH free radical scavenging assay is a reliable and useful in vitro method to measure antioxidative activities of phytoconstituents in plant extracts (Anu et al. 2025; Diment et al. 2025). This assay is based on the principle of a stable free radical compound, 1,1‐diphenyl‐2‐picrylhydrazyl (DPPH•), which has a deep violet color and high absorbance at 517 nm. When an antioxidant donates a hydrogen atom to DPPH•, it becomes reduced to DPPH‐H, a pale yellow, non‐radical form. Consequently, a decrease in absorbance is observed, which reflects the antioxidant capacity of the test samples. Thus, the free radical scavenging capacity of the AiME was evaluated using a modified version of this established DPPH (1,1‐diphenyl‐2‐picrylhydrazyl) radical scavenging assay (Alhakmani et al. 2013). Briefly, a 0.1 mM DPPH solution in absolute methanol was prepared fresh and kept in the dark at 4°C during the experiment. The AiME extract was serially diluted to various concentrations (10–500 μg/mL) using methanol. Subsequently, 0.3 mL aliquots of each AiME extract dilution were added to 3 mL aliquots of the DPPH solution. The reaction mixtures were incubated for 30 min in the dark to allow for reaction, followed by absorbance measurement at 517 nm. Methanol served as a blank control, whereas ascorbic acid was employed as a standard and processed identically to the plant extract. All experiments were conducted in triplicate, and the results are presented as the average value.

$$\%\text{Of radical scavenging capacity}=\left[\left(A_{0}-A_{t}\right)/A_{0}\times 100\right]$$

where *A*_0_ = absorbance value of blank sample; *A*_*t*_ = absorbance value of the test samples.

#### 2.2.2 Reducing Power Capability Assay

Reducing power capability is another antioxidant assay widely used to determine the overall electron‐donating ability in plant extracts (Gulcin and Alwasel 2025; Irshad et al. 2025). In this assay, the principle is the ability of test sample compounds to reduce ferric ions (Fe^3+^) to ferrous ions (Fe^2+^), a crucial parameter to assess the antioxidant activity in test samples. Therefore, the ferric reducing activity (FRA) of AiME was determined by its ability to convert ferric ions (Fe^3+^) to ferrous ions (Fe^2+^) following the method established (Anandjiwala et al. 2008). Briefly, varying concentrations (10–500 μg/mL) of the plant extract (AiME) were incubated with 2.5 mL of phosphate buffer (pH 6.6) containing 1% potassium ferricyanide (2.5 mL) at 50°C 500C for 20 min. The reaction was terminated by the addition of trichloroacetic acid (2.5 mL) and subsequent centrifugation at 3000 rpm for 10 min. The supernatant (2.5 mL) was then mixed with distilled water (2.5 mL) and freshly prepared ferric chloride solution (FeCl_3_, 0.1%, 0.5 mL). Absorbance of the final mixture was measured at 700 nm. Ascorbic acid served as a positive control, undergoing the same treatment as the samples. Increased absorbance values reflected greater reducing power of the extract. Each experiment was performed in triplicate, and the results were averaged for analysis.

#### 2.2.3 Ferric‐Reducing Antioxidant Power (FRAP) Assay

The ferric reducing ability of the AiME was evaluated following a previously reported method by Benzie and Strain (1996). This modification involved the preparation of a FRAP reagent by combining 100 mL of 300 mM acetate buffer (pH 3.6), 10 mL of 10 mM TPTZ solution (in 40 mM hydrochloric acid), and 10 mL of 20 mM FeCl3.6H2O. The freshly prepared FRAP reagent was warmed to 37°C prior to use. Subsequently, 0.3 mL of various concentrations (10–500 μg/mL) of the plant extract (AiME) was added to 3 mL of the FRAP reagent in a test tube. The mixture was then incubated in darkness for 4 min, followed by absorbance measurement at 593 nm. Ascorbic acid served as a positive control, undergoing the same treatment as the plant extract samples.

### 2.3 In Vitro Acetylcholinesterase Assay

A microplate assay with minor modifications to Ellman's method (Ellman et al. 1961) was employed to assess the AiME's potential to inhibit AChE activity. Minor modifications included varying plant extract concentrations, using 405 nm instead of 412 nm, and adding DMSO as a negative control, which did not affect the evaluation of anti‐acetylcholinesterase activity. Briefly, a range of different concentrations of plant extract AiME (ranging from 0.05 to 10 mg/mL) were prepared through serial dilution and dispensed into quadruplicate wells of a microplate. After adding AiME extract to each Eppendorf tube, following the addition of extracts, each well received 50 μL of phosphate buffer (containing 8 mM K_2_HPO_4_, 2.3 mM NaH_2_PO_4_, and 0.15 M NaCl, pH 7.6) and 50 μL (0.25 U/mL) AChE enzyme from *Electrophorus electricus* (Sigma‐Aldrich, USA) in phosphate buffer, followed by a 20‐min incubation at 20°C. Afterwards, 25 μL of ATCI (acetylthiocholine iodide, 5.18 mg/mL) and 125 μL of 5,5′‐Dithiobis (2‐nitrobenzoic acid) (DTNB, 0.3 mg/mL) were added to each well, and the mixture was incubated for another 20 min at 37°C, leading to the formation of a yellow substance. The absorbance of this yellow substance, transferred to a 96‐well ELISA plate (*A*_sample_), was measured at 405 nm using a UV spectrophotometer. Control wells included a sample blank (*A*_Blank_) lacking AChE and the sample, and a control (*A*_control_) containing all reagents except the sample. The validity of the assay was established by comparing the control to Rivastigmine, a known AChE inhibitor. The percentage inhibition of AChE by the *A. indica* extract was calculated using the following equation:

$$\%\text{Of AChE inhibition}=\left(1-A_{\text{sample}}/A_{\text{control}}\right)\times 100$$

### 2.4 In Vivo Anti‐Inflammatory Study

#### 2.4.1 Carrageenan Treatment

A total of 24 mice of both sexes were allocated into four groups of six animals each, which were bred and housed at the International Centre for Diarrheal Disease and Research in Bangladesh (ICDDRB). Figure S1, depicts paw size changes in mice during the carrageenan treatment. The animals were kept in standard laboratory conditions, which included a relative humidity of 55%–65%, ambient temperature of 25°C–28°C, and 12‐h light–dark cycles. Animals were provided with a continuous availability of food, pellets and water. Animals were divided into the following four groups: negative control, treatment group, AiME extract at 200 mg/kg, and AiME extract at 400 mg/kg. Then, a single dose of normal saline (1 mL/100 g), indomethacin 10 mg/kg, AiME 200 mg/kg, and AiME 400 mg/kg were administered orally (p.o.) to the four groups of animals, respectively. After 30 min, paw edema was induced in each mouse by injecting 0.1 mL of a 1% carrageenan solution into the plantar region of the right hind paw. All experimental procedures were conducted in compliance with the ARRIVE guidelines (Percie du Sert et al. 2020). The study protocol was approved by the institutional ethics committee under reference number (40/2020).

#### 2.4.2 Paw Size Measuring

Paw thickness was assessed at baseline using a Vernier caliper before carrageenan injection. Subsequently, paw thickness was measured at following intervals (1, 2, 3, and 4 h) after carrageenan injection to monitor edema development. The change in paw thickness was calculated as the difference between the baseline measurement and the respective hourly measurements.

### 2.5 LCMS/MS Analysis

In the present study, a quantitative evaluation of 56 standards (53 phytochemicals and 3 deuterated internal standards) was conducted using a Shimadzu Nexera UHPLC system coupled to a tandem mass spectrometer, following the method of Yilmaz (2020). The UHPLC system consisted of an autosampler (SIL‐30AC), a column oven (CTO‐10ASvp), binary pumps (LC‐30AD), and a degasser (DGU‐20A3R). Chromatographic separation was achieved using an Agilent Poroshell 120 EC‐C18 analytical column (150 mm × 2.1 mm, 2.7 μm) maintained at 40°C. The mobile phases consisted of eluent A (water with 5 mM ammonium formate and 0.1% formic acid) and eluent B (methanol with 5 mM ammonium formate and 0.1% formic acid). The gradient profile was: 20%–100% B (0–25 min), 100% B (25–35 min), and return to 20% B (35–45 min). The flow rate and injection volume were set at 0.5 mL/min and 5 μL, respectively. Prior to LC–MS/MS analysis, extracts were diluted with methanol to a final concentration of 1 mg/mL and maintained at 15°C during analysis (Yilmaz 2020). The File S1A provides details about LC–MS/MS analysis. The LC–MS/MS chromatogram of 53 standard compounds and 3 internal standards is given in Figure S2.

### 2.6 GCMS Analysis

The tentative identification of *A. indica* plant constituents was achieved using a Shimadzu GC–MS QP 2020 equipped with an SH‐Rxi‐% Sil MS packed fused silica column, as explained by Settu et al. (2021). Helium served as carrier gas with a constant flow rate of 1 mL/min. The injector temperature was set to 250°C, and a 1 μL sample was injected. The temperature program employed an initial hold at 50°C for 4 min, followed by a rapid temperature increase of 8°C/min to reach a final temperature of 280°C, which was maintained for 2 min. The mass spectrometry (MS) detector operated with a transfer line temperature of 250°C, an ion source temperature of 200°C, and electron ionization (EI) mode at 70 eV. The scan parameters included a scan time of 0.30 s and a scan interval of 0.1 s, acquiring fragments within a mass range of 40–550 Da. The acquired mass spectra were then compared to those of known compounds within the GC–MS NIST library for identification purposes.

### 2.7 In Silico Analysis

#### 2.7.1 Molecular Docking Study

Molecular docking simulations were performed using the AutoDock Vina software, following the same protocols described in our previous study. The key residues in the active site of AChE (PDB ID: 4EY7) were identified as Tyr72, Trp86, Trp286, Ser293, Phe295, Tyr337, Phe338, and Tyr341. These residues are potentially responsible for regulating AChE physiology and activity (Jiang and Gao 2018; Kumar et al. 2024; Roca et al. 2018). A ligand library of 50 compounds was compiled using data from LC–MS/MS, GC–MS, and previously reported literature sources outlined in Table S2 (Ho et al. 2004, 2003). The reference drug rivastigmine was also included for validation. Detailed information on protein and ligand preparation, as well as grid box coordinates, is provided in File S1A.

### 2.8 Drug‐Likeness and ADMET Properties Screening

After a successful molecular docking, 12 candidate compounds derived from AiME were chosen for further analysis. This analysis evaluated their drug‐likeness properties and adherence to Lipinski's rule of five (“RO5”). The SwissAdme platform (http://www.swissadme.ch/) was utilized to compute drug‐likeness scores based on the established Lipinski rule (Jain et al. 2024). Additionally, bioavailability radars were generated for all compounds, allowing for a visual comparison of their physicochemical properties to the reference drug, rivastigmine (Daina et al. 2017). Furthermore, pkCSM (https://biosig.lab.uq.edu.au/pkcsm/) was employed to assess a comprehensive range of ADMET (absorption, distribution, metabolism, excretion, and toxicity) properties (de Araújo et al. 2025). The inclusion of rivastigmine in both analyses served as a control, enabling the standardization of predicted values for the proposed ligands by facilitating a direct comparison with a known drug for AD.

### 2.9 Molecular Dynamics (MD) Simulations

Molecular dynamics simulations of 100 ns were conducted to examine the apoenzyme AChE in complex with the lead compounds acteoside and acacetin, following the methodology outlined in our previous study (Ahmed et al. 2024; Mazumder et al. 2022). Structural parameters, including root‐mean‐square deviation (RMSD), root‐mean‐square fluctuation (RMSF), radius of gyration (Rg), solvent‐accessible surface area (SASA), and hydrogen bond interactions, were analyzed for both complexes. Stepwise details are provided in File S1A.

### 2.10 Statistical Analysis

All data are expressed as the mean ± SEM. Statistical analyses were performed using GraphPad Prism (version 8.3.4). For the antioxidant assay, both standard and plant extract groups were analyzed by a two‐way ANOVA test. In the anti‐inflammatory assay, ANOVA was followed by Bonferroni's test. A probability level (adjusted *p*‐value calculated by GraphPad Prism) of 0.05 or less was considered significant; *p < 0.05, **p < 0.01, ***p < 0.001.

## 3 Results

### 3.1 In Vitro Study

#### 3.1.1 Results of Antioxidant Assay for AiME

**FIGURE 1:**
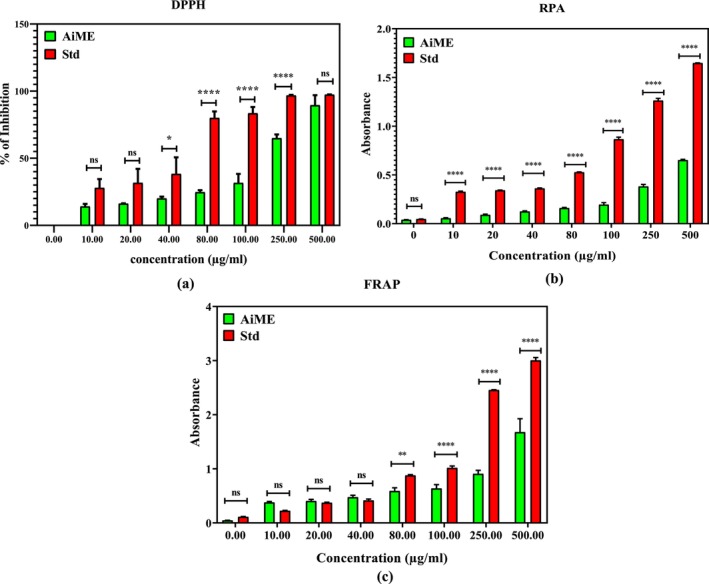
Graphical representations for antioxidant activity of *Aeginetia indica* ; (a) comparison of % of inhibition results between *A. indica* and ascorbic acid in DPPH free radical scavenging activity, (b) result of reducing power activity (RPA), (c) ferric‐reducing antioxidant power (FRAP); AiME: methanolic extract of *A. indica* ; Std: ascorbic acid.

This study aimed to investigate the antioxidant potential of AiME and elucidate its underlying mechanism. Three assays, DPPH free radical scavenging activity, reducing power activity (RPA), and ferric‐reducing antioxidant power (FRAP) assay, were employed to assess the antioxidant properties of AiME (Figure 1a–c). Additionally, comparisons of IC_50_ and EC_50_ values between AiME and ascorbic acid (Std) are summarized in Table 1.

**TABLE 1:** Comparison of IC_50_ and EC_50_ values between AiME and ascorbic acid (Std) during in vitro antioxidant assay.

Assay	Sample (*N* = 3)	IC_50_ value (μg/mL)
DPPH free radical scavenging activity	AiME	176.586 ± 5.960
	Ascorbic acid (Std)	38.866 ± 10.059

Assay	Sample	EC_50_ (μg/mL)
Reducing power activity (RPA)	AiME	358.249 ± 16.605
	Ascorbic acid (Std)	53.778 ± 0.624
Ferric‐reducing antioxidant power (FRAP)	AiME	39.467 ± 9.046
	Ascorbic acid (Std)	48.986 ± 0.512

The DPPH free radical scavenging assay revealed a similar trend of increasing activity with concentration for both AiME and standard ascorbic acid (Figure 1a). However, ascorbic acid exhibited a significantly stronger free radical scavenging ability, with an IC_50_ value of 38.866 ± 10.059 μg/mL compared to AiME's 176.586 ± 5.96 μg/mL (Table 1). The RPA, another indicator of antioxidant potential, was measured by the increase in absorbance at 700 nm. AiME displayed a lower reducing power (RPA) compared to ascorbic acid (Std), reaching an average maximum absorbance of 0.652 at 500 μg/mL (Figure 1b). In contrast, standard ascorbic acid exhibited an average maximum absorbance of 1.648 at 500 μg/mL. This trend mirrored the observations from the DPPH assay. The FRAP assay, another antioxidant assay, demonstrated the ferric‐reducing antioxidant power activity in AiME compared to the standard. The average maximum absorbance for AiME was 1.679 at a concentration of 500 μg/mL, whereas ascorbic acid (Std) had an average maximum value of 3.006 at the same concentration (Figure 1c).

#### 3.1.2 Results of In Vitro AChE Inhibition Assay for AiME

**FIGURE 2:**
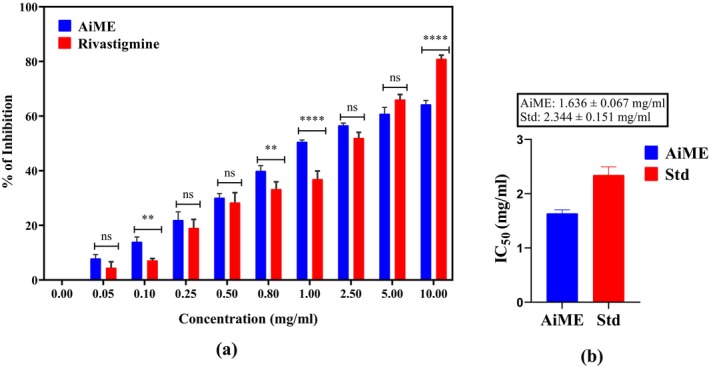
Comparison of percentage of inhibition results and IC_50_ between *Aeginetia indica* and rivastigmine (Std) for AChE inhibition; AiME: methanolic extract of *A. indica* ; Std: ascorbic acid.

Figure 2 summarizes the inhibitory activity of *A. indica* methanolic extract (AiME) against AChE and the corresponding IC_50_ values. Rivastigmine, a known AChE inhibitor, was used as the standard for comparison. Both AiME and rivastigmine exhibited concentration‐dependent inhibition (Figure 2a). Notably, AiME demonstrated a stronger inhibitory effect than the standard. The IC_50_ value of AiME was 1.636 ± 0.067 mg/mL, whereas rivastigmine (standard) showed an IC_50_ of 2.344 ± 0.151 mg/mL (Figure 2b). The lower IC_50_ value of AiME suggests potentially greater AChE inhibitory activity compared to rivastigmine.

### 3.2 In Vivo Anti‐Inflammatory Study

AiME demonstrated anti‐inflammatory efficacy at all provided doses, as detailed in Table 2. The study demonstrates the anti‐inflammatory effects of indomethacin (Std) and the AiME on paw edema in an experimental model. Indomethacin, a known nonsteroidal anti‐inflammatory drug, inhibited paw edema by 26.84% at the 4‐h mark, serving as a standard for comparison. In comparison with Std, our plant extract AiME at a dose of 400 mg/kg showed nearly an equivalent inhibition of 26.81% at the end of 4 h, suggesting it has similar efficacy to indomethacin at this dosage (400 mg/kg). However, at a lower dose of 200 mg/kg, AiME inhibited paw edema by 20.35% at the 4‐h mark, demonstrating a dose‐dependent response. The similar efficacy trend of edema inhibition observed in both AiME and the standard treatment is supported by a highly significant statistical difference, with *p* < 0.001 (***) between the two groups.

**TABLE 2:** The effects of the methanol extract of *Aeginetia indica* and indomethacin in carrageenan‐induced paw edema test in mice.

Treatment group	Paw size					Total increase or decrease of paw thickness
	At 0 h	After 1 h	After 2 h	After 3 h	After 4 h	
Negative control	2.34 ± 0.02	3.58 ± 0.05	3.57 ± 0.11	3.43 ± 0.06	3.39 ± 0.07	44.87% ↑
Standard indomethacin	2.38 ± 0.06	2.99 ± 0.09***	2.73 ± 0.10***	2.68 ± 0.05***	2.48 ± 0.06***	26.84% ↓
*A. indica* 200 mg/kg	2.43 ± 0.03	3.1 ± 0.14***	2.95 ± 0.10***	2.76 ± 0.08***	2.70 ± 0.06***	20.35% ↓
*A. indica* 400 mg/kg	2.37 ± 0.02	2.83 ± 0.08***	2.7 ± 0.10***	2.63 ± 0.09***	2.48 ± 0.07***	26.81% ↓

*Note:* (↑) indicate percentage increase of paw edema size after 4 h compared to 0 h. (↓) indicate percentage decrease of paw edema size after 4 h compared to negative control group. All values are given in mean ± SEM. *N* = 6. Significantly different from negative control at *p < 0.05, **p < 0.01, and ***p < 0.001.

### 3.3 Phytochemical Study

#### 3.3.1 Results From LCMS Analysis

**FIGURE 3:**
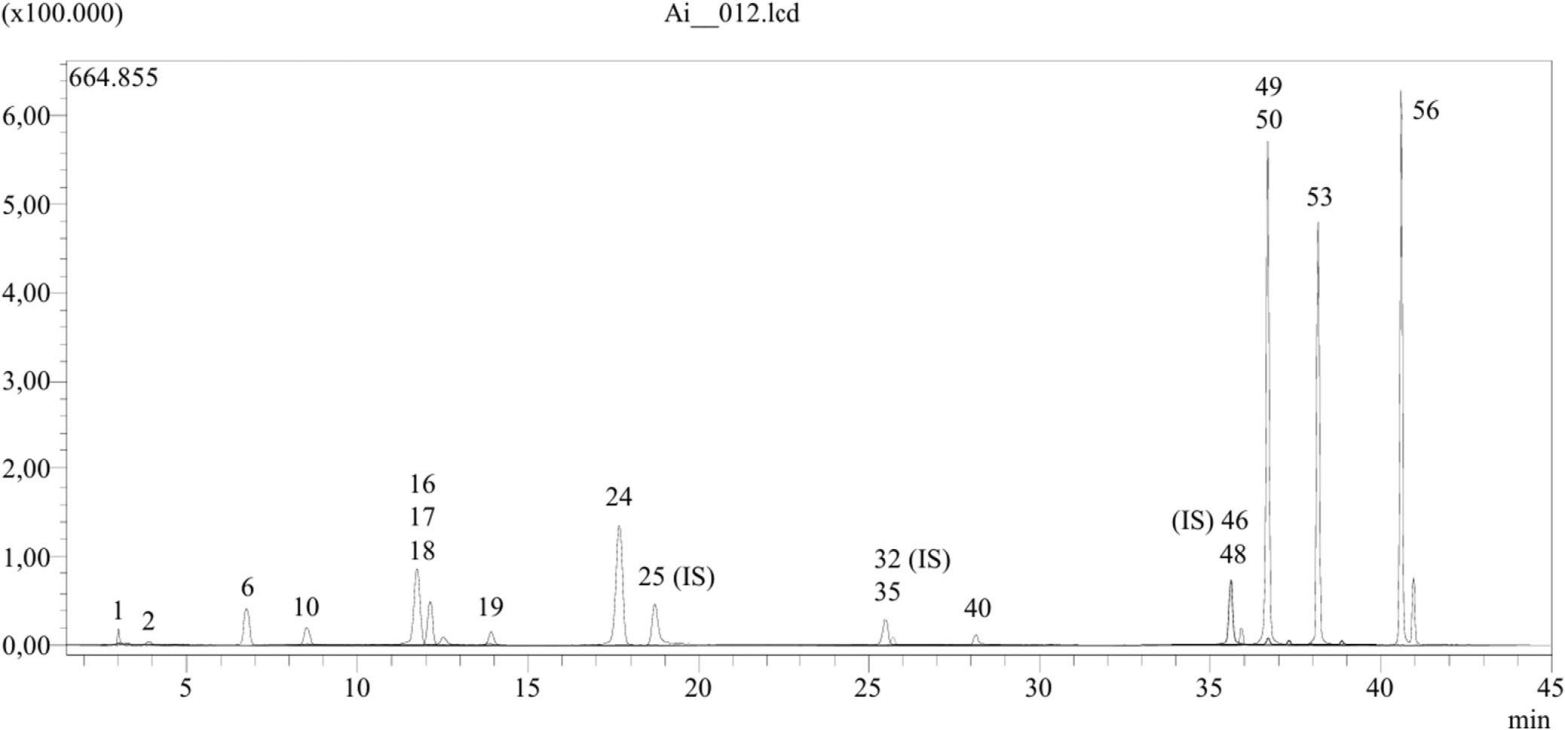
LC–MS/MS chromatogram of *Aginetia indica* whole plant extract.

The polyphenolic composition of the AiME was analyzed by LC–MS/MS analysis, and the findings are presented in Table 3. This analysis identified 16 polyphenolic compounds in the AiME extract through comparison with standards. The corresponding chromatogram for AiME is presented in Figure 3, whereas the LC–MS/MS chromatogram of the standard compounds is provided in Figure S2. The overall analytical method validation parameters employed by this study are also summarized in Table S1. Quantitative analysis revealed that the four most abundant polyphenols in AiME were acacetin (2.972 mg/g), fumaric acid (2.544 mg/g), vanilic acid (1.926 mg/g), and luteolin (1.460 mg/g). Other major identified compounds included protocatechuic acid (1.449 mg/g), apigenin (0.895 mg/g), and caffeic acid (0.652 mg/g). Protocatechuic aldehyde, quinic acid, vanillin, syringic acid, and p‐coumaric acid were all found in lower amounts of 0.379 mg/100 g, 0.332, 0.294, and 0.122 mg/g, respectively. The phenolics with the lowest abundances were naringenin (0.081 mg/g), hesperetin (0.037 mg/g), hesperidin (0.024 mg/g), and cosmosin (0.023 mg/g). Compounds such as acacetin, luteolin, caffeic acid, and vanillic acid are known to inhibit lipid oxidation and oxidative degradation in food matrices, whereas flavonoids like apigenin and naringenin have been associated with functional food development owing to their potential health‐promoting effects (Parveen et al. 2025). The abundance of diverse polyphenolic compounds in AiME suggests strong natural antioxidants, anti‐inflammatory potential, which may help reduce oxidative stress in the body when consumed as a dietary supplement (Sun et al. 2024). Moreover, its enriched polyphenolic profile highlights the significance of this herbal plant for the food and beverage industries, particularly for its potential use as an anti‐browning agent (Ho 1992). Collectively, the diverse polyphenolic profile of AiME supports its potential application as a natural functional ingredient or nutraceutical additive in food systems (Martínez‐Zamora et al. 2023).

**TABLE 3:** Identification and quantification of phenolic compounds of *Aeginetia indica* by LC–MS/MS.

Analytes	AiME (mg analyte/g extract)	R.T.	MI (*m*/*z*)	FI (*m*/*z*)
Quinic acid	0.323	3.0	190.8	93.0
Fumaric acid	2.544	3.9	115.2	40.9
Aconitic acid	N.D.	4.0	172.8	129.0
Gallic acid	N.D.	4.4	168.8	79.0
Epigallocatechin	N.D.	6.7	304.8	219.0
Protocatechuic acid	1.449	6.8	152.8	108.0
Catechin	N.D.	7.4	288.8	203.1
Gentisic acid	N.D.	8.3	152.8	109.0
Chlorogenic acid	N.D.	8.4	353.0	85.0
Protocatechuic aldehyde	0.379	8.5	137.2	92.0
Tannic acid	N.D.	9.2	182.8	78.0
Epigallocatechin gallate	N.D.	9.4	457.0	305.1
1,5‐dicaffeoylquinic acid	N.D.	9.8	515.0	191.0
4‐OH benzoic acid	N.D.	10.5	137.2	65.0
Epicatechin	N.D.	11.6	289.0	203.0
Vanillic acid	1.926	11.8	166.8	108.0
Caffeic acid	0.652	12.1	179.0	134.0
Syringic acid	0.294	12.6	196.8	166.9
Vanillin	0.332	13.9	153.1	125.0
Syringic aldehyde	N.D.	14.6	181.0	151.1
Daidzin	N.D.	15.2	417.1	199.0
Epicatechin gallate	N.D.	15.5	441.0	289.0
Piceid	N.D.	17.2	391.0	135/106.9
*p‐*coumaric acid	N.D.	17.8	163.0	93.0
Ferulic acid‐D3‐IS*	N.A.	18.8	196.2	152.1
Ferulic acid	N.D.	18.8	192.8	149.0
Sinapic acid	N.D.	18.9	222.8	193.0
Coumarin	N.D.	20.9	146.9	103.1
Salicylic acid	N.D.	21.8	137.2	65.0
Cynaroside	N.D.	23.7	447.0	284.0
Miquelianin	N.D.	24.1	477.0	150.9
Rutin‐D3‐IS*	N.A.	25.5	612.2	304.1
p‐Coumaric acid	0.122	17.8	163.0	93.0
Hesperidin	0.024	25.8	611.2	449.0
*o*‐Coumaric acid	N.D.	26.1	162.8	93.0
Genistin	N.D.	26.3	431.0	239.0
Rosmarinic acid	N.D.	26.6	359.0	197.0
Ellagic acid	N.D.	27.6	301.0	284.0
Cosmosin	0.023	28.2	431.0	269.0
Quercitrin	N.D.	29.8	447.0	301.0
Astragalin	N.D.	30.4	447.0	255.0
Nicotiflorin	N.D.	30.6	592.9	255.0/284.0
Fisetin	N.D.	30.6	285.0	163.0
Daidzein	N.D.	34.0	253.0	223.0
Quercetin‐D3‐IS*	N.D.	35.6	304.0	275.9
Quercetin	N.D.	35.7	301.0	136.0/286.0
Naringenin	0.081	35.9	270.9	119.0
Hesperetin	0.037	36.7	301.0	136.0/286.0
Luteolin	1.460	36.7	284.8	151.0/175.0
Genistein	N.D.	36.9	269.0	135.0
Kaempferol	N.D.	37.9	285.0	239.0
Apigenin	0.895	38.2	268.8	151.0/149.0
Amentoflavone	N.D.	39.7	537.0	417.0
Chrysin	N.D.	40.5	252.8	145.0/119.0
Acacetin	2.972	40.7	283.0	239.0

Abbreviations: FI (*m*/*z*), fragment ions; MI (*m*/*z*), molecular ions of the standard analytes (*m*/*z* ratio); N.D., not detected; NA, not applicable; R.T., retention time.

#### 3.3.2 Results From GCMS Analysis

**FIGURE 4:**
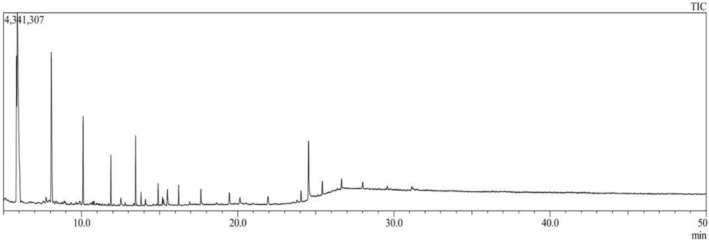
GCMS chromatogram for *Aginetia indica* whole plant extract.

GC–MS analysis was employed to tentatively identify potential phytochemical compounds in the AiME. The characteristic GC–MS chromatogram of AiME is presented in Figure 4. A total of 23 compounds were identified in the AiME and are listed in Table 4. This table includes the retention time, molecular weight, molecular formula, percentage area, and other relevant information for each compound identified by GC–MS analysis.

**TABLE 4:** List of identified polyphenolic compounds from the methanolic extract of *Aeginetia indica* via GCMS analysis.

Sl. no.	Compound ( *A. indica* )	R. time	*m*/*z*	MW (g/mol)	Molecular formula	Area
1	2H‐1,4‐Benzodiazepin‐2‐one, 7‐chloro‐1,3‐dih	5.835	73	310.48	C_19_H_34_O_3_	64,119
2	Benzeneacetic acid, 3‐nitro—	7.721	73	154.20	C_9_H_14_O_2_	64,119
3	Galacto‐heptulose	8.046	67	294.50	C_19_H_34_O	40,434
4	Phloroglucinol	9.103	74	340.60	C_22_H_44_O_2_	135,320
5	1,2‐Benzenedicarboxylic acid, butyl octyl ester	9.854	74	214.34	C_13_H_26_O_2_	345,315
6	Tetrahydro‐4H‐pyran‐4‐ol	10.566	74	298.50	C_19_H_38_O_2_	345,315
7	Gamma‐Guanidinobutyric acid	10.925	59	281.50	C_18_H_35_NO	350,171
8	Methoprene	12.519	73	258.43	C_13_H_26_O_3_Si	64,119
9	Phthalic acid, butyl ester, ester with butyl glycol	12.796	74	284.50	C_18_H_36_O_2_	135,320
10	Methyl tetradecanoate	13.463	59	309.53	C_20_ H_39_NO	350,171
11	Bicyclo[3.1.0]hexane‐2‐undecanoic acid, methyl ester	14.091	73	320.50	C_21_H_36_O_2_	47,441
12	12,15‐Octadecadienoic acid, methyl ester	15.186	74	284.50	C_18_H_36_O_2_	345,315
13	Linoelaidic acid	15.186	73	310.48	C_17_H_32_O_4_Si_3_	47,441
14	10‐Undecyn‐1‐ol	15.35	44	145.16	C_5_H_11_N_3_O_2_	20,481
15	Docosanoic acid, methyl ester	15.494	73	266.50	C_18_H_34_O	47,441
16	Trimethylsilyl 3‐methyl‐4‐ [(trimethylsilyl)oxy]	16.932	74	242.40	C_15_H_30_O_2_	345,315
17	Dodecanoic acid, 2,3‐bis (acetyloxy)propyl ester	18.497	67	280.44	C_18_H_32_O	40,434
18	2,5‐Dihydroxybenzoic acid, 3TMS derivative	19.466	74	270.45	C_17_H_34_O	345,315
19	Octadecanoic acid, 2,3‐dihydroxypropyl ester	19.92	73	310.48	C_18_H_37_NO_3_Si_3_	47,441
20	1,2‐Ethanediamine, N‐(2‐aminoethyl)—	20.683	44	198.30	C_12_H_22_O_2_	8078
21	Cyclopentadecanone, oxime	23.394	73	294.47	C_19_H_34_O	47,441
22	9‐Octadecenamide, (Z)—	24.536	44	184.28	C_11_H_20_O_2_	8078
23	7,7,9,9,11,11‐Hexamethyl‐3,6,8,10,12,15‐hex	29.61	73	322.50	C_21_H_38_O_2_	47,441

### 3.4 Molecular Docking Analysis

A total of 45 compounds, including the reference standard rivastigmine, were docked against AChE to evaluate their binding affinities. Table 5 summarizes the results in ascending order. Notably, 19 AiME‐derived compounds displayed superior binding affinities (−10.9 to −8.2 kcal/mol) compared to rivastigmine (−8.1 kcal/mol). Hesperidin emerged as the most promising candidate with the strongest binding affinity (−10.9 kcal/mol). Several other compounds, including aegineoside, acacetin, and luteolin, also demonstrated promising potential with affinities exceeding −10.0 kcal/mol. Furthermore, acteoside, ficusal, cosmosin, aeginetic acid, and aeginetolide exhibited affinities (−9.6 to −8.5 kcal/mol) surpassing rivastigmine.

**TABLE 5:** Docking score of identified phytochemical compounds and standard drug (rivastigmine) in the binding site of acetylcholinesterase (4ey7) computed via Autodoc Vina Software.

Sl. no.	Ligand	CID	Binding affinity (kcal/mol)
1	Hesperidin	10,621	−10.9
2	Aegineoside	132,353,271	−10.7
3	Acacetin	5,280,442	−10.4
4	Hesperetin	72,281	−10.4
5	Luteolin	5,280,445	−10.4
6	Naringenin	439,246	−10.3
7	Stigmaserol	5,280,794	−10.2
8	Apigenin	5,280,443	−10.2
9	Balanophonin	23,252,258	−10.1
10	Beta‐sitosterol	222,284	−10.0
11	Acteoside	5,281,800	−9.6
12	Ficusal	10,496,641	−9.0
13	Cosmosin	6,336,505	−8.7
14	Aeginetic acid	15,693,867	−8.7
15	1,2‐Benzenedicarboxylic‐acid‐butyl‐octyl‐ester	66,540	−8.6
16	Bicyclo[3.1.0]hexane‐2‐undecanoic acid, methyl ester	557,192	−8.5
17	Aeginetolide	15,948,056	−8.5
18	Cyclopentadecanone oxime	551,985	−8.4
19	Methoprene	5,366,546	−8.2
20	Rivastigmine	77,991	−8.1
21	9‐Octadecenamide, E	5,362,793	−7.7
22	Benzeneacetic acid, 3‐nitro—	15,876	−7.7
23	Linoelaidic acid	5,282,457	−7.7
24	Caffeic acid	689,043	−7.7
25	Octadecanoic acid, 2,3‐dihydroxypropyl ester	24,699	−7.6
26	p‐Coumaric acid	637,542	−7.6
27	12,15‐Octadecadienoic acid, methyl ester	5,365,571	−7.4
28	Methyl tetradecanoate	31,284	−7.3
29	β‐sitosteryl glucoside	5,742,590	−7.1
30	Vanillic acid	8468	−6.8
31	2‐Dodecenoic acid	5,282,729	−6.7
32	Quinic acid	6508	−6.6
33	Protocatechuic acid	72	−6.6
34	Syringic acid	10,742	−6.6
35	10‐Undecyn‐1‐ol	76,015	−6.4
36	Protocatechuic aldehyde	8768	−6.2
37	Vanillin	1183	−6.2
38	Galacto‐heptulose	102,926	−5.7
39	Gamma‐Guanidinobutyric acid	500	−5.6
40	Phloroglucitol	230,351	−5.2
41	β‐sitosteryl glucoside	5,742,590	−5.0
42	Trans‐coniferaldehyde	5,280,536	−4.8
43	Fumaric acid	444,972	−4.6
44	Tetrahydro‐4H‐pyran‐4‐ol	74,956	−4.4
45	1,2‐Ethanediamine‐N‐(2‐aminoethyl)—	8111	−4.0

**FIGURE 5:**
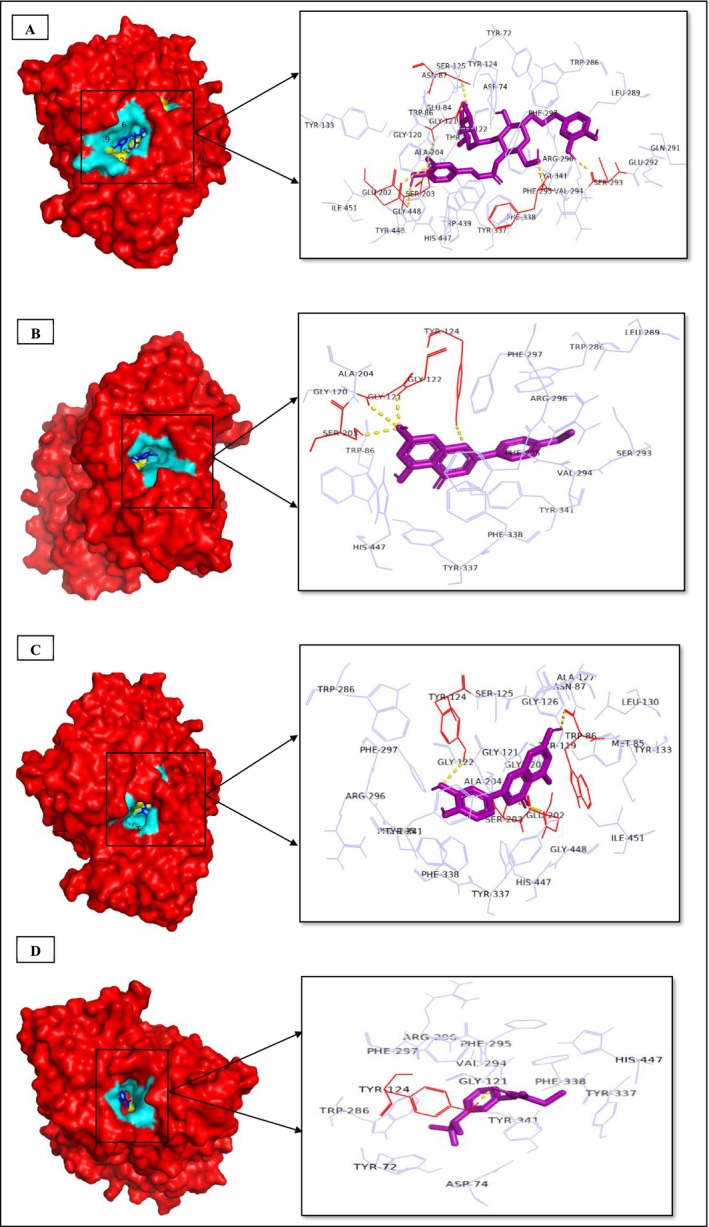
Possible 3D interactions of (A) acteoside, (B) acacetin, (C) luteolin, (D) rivastigmine with acetylcholinesterase (AChE) (PDB ID: 4EY7) (pose predicted by PyMoL).

Based on these findings, 12 compounds were selected as potential “hit” candidates for AChE inhibition, considering their abundance in AiME and promising docking scores, and previous histories of association with acetylcholinesterase activity. The three best “lead” compounds (acteoside, acacetin, and luteolin) were further analyzed for their bonding interactions with AChE, presented in Table 6. Similar analyses for the remaining 9 hit compounds are provided in Table S3. Notably, the three lead compounds displayed equal or more hydrogen bonds with AChE compared to rivastigmine. To comprehensively understand the protein‐ligand interactions, 2d representations for all hit compounds are provided in Figure S3. Additionally, 3d structural representations in Figure 5 and Figure S4 visually depict these interactions for all selected lead candidates.

**TABLE 6:** Bond analysis among the three lead phytoconstituents and rivastigmine (standard) with acetylcholinesterase enzyme (AchE).

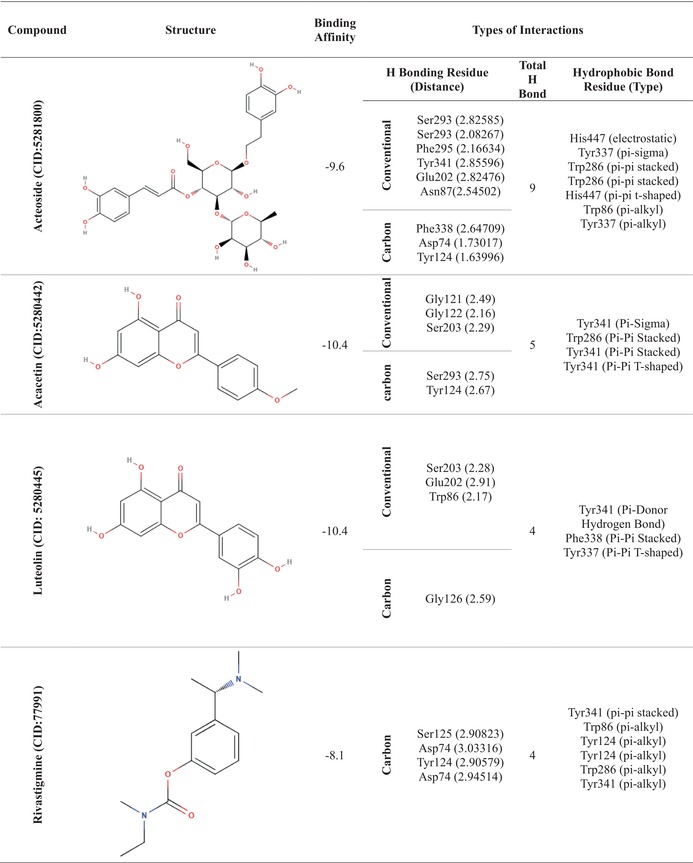

### 3.5 Drug Likeliness, ADMET, Bioavailability Radar Analysis

Twelve hit compounds were assessed for drug‐likeness and ADMET properties (summarized in Tables 7 and 8). All compounds except acteoside, hesperidin, and aeginetolide adhered to Lipinski's Rule (Jain et al. 2024). Additionally, all compounds, except them, displayed high value for the GI absorption. Despite acteoside's limited water solubility (MLOGP < 5), it was retained due to potential mitigation strategies such as chitosan‐coated liposomes described by Xiao et al. (2022). Interestingly, both acacetin and luteolin, among the selected three best lead compounds, exhibited a TPSA value within the optimal range of 20–130 Å^2^, suggesting good potential for crossing biological barriers, such as absorption and brain access, which is important for AD drugs (Gandla et al. 2023).

**TABLE 7:** Evaluation of drug likeliness properties of nine lead compounds and rivastigmine by examining Lipinski rules violations.

Compound	Molecular formula	MW (g/mol)	NHA	NHD	TSPA	GI absorption	Log Po/w (MLOGP)	Lead likeness	Lipinski violations
Acteoside	C_29_H_36_O_15_	624.59	15	9	245.29	Low	−2.37	No: 2 violations	3 violations
Hesperidin	C_28_H_34_O_15_	610.56	15	8	234.29	Low	−3.04	No: 1 violation	3 violations
Acacetin	C_16_H_12_O_5_	284.26	5	2	79.90	High	0.77	Yes	0 violation
Hesperetin	C_16_H_14_O_6_	302.28	6	3	96.22	High	0.41	Yes	0 violation
Luteolin	C_15_H_10_O_6_	286.24	6	4	111.13	High	−0.03	Yes	0 violation
Naringenin	C_15_H_12_O_5_	272.25	5	3	86.99	High	0.71	Yes	0 violation
Aegineoside	C_26_H_30_O_12_	534.51	12	6	184.60	Low	−1.15	No: 2 violations	3 violations
Aeginetic acid	C_15_H_24_O_4_	268.35	4	3	77.76	High	1.62	Yes	0 violation
Aeginetolide	C_11_H_18_O_3_	198.26	3	1	46.53	High	1.60	No: 1 violation	0 violation
Apigenin	C_15_H_10_O_5_	270.24	5	3	90.90	High	0.52	Yes	0 violation
Balanophonin	C_20_H_20_O_6_	356.37	6	2	85.22	High	1.01	No: 1 violation	0 violation
Ficusal	C_18_H_18_O_6_	330.33	6	2	85.22	High	0.63	Yes	0 violation
Rivastigmine	C_14_H_22_N_2_O_2_	250.34	6	3	32.78	High	2.34	Yes	0 violation

**TABLE 8:** ADMET properties analysis of the best 10 compounds with rivastigmine (standard).

Parameter	Acteoside	Selected phytoconstituents												STD	Reference value
		Hesperidin	Acacetin	Hesperetin	Luteolin	Naringenin	Aegineoside	Aeginetic acid	Aeginetolide	Apigenin	Balanophonin	Ficusal	Rivastigmine		
Absorption	Water solubility (log mol/L)	−2.90	−3.01	−3.28	−3.04	−3.09	−3.22	−3.40	−1.64	−2.04	−3.32	−4.24	−3.72	−2.35	—
	Caco2 permeability	0.09	0.50	1.13	0.29	0.09	1.02	−0.63	0.88	1.23	1.00	1.27	1.26	1.57	High: > 0.90
	Intestinal absorption	32.11	31.48	94.31	70.27	81.13	91.31	20.82	94.18	95.21	93.25	96.04	95.83	88.46	Poor: < 30%
	Skin permeability	−2.73	−2.73	−2.73	−2.73	−2.73	−2.74	−2.73	−2.73	−3.58	−2.73	−2.75	−2.82	−2.88	Low: > −2.5
	P‐glycoprotein substrate	Yes	Yes	Yes	Yes	Yes	Yes	Yes	No	No	Yes	Yes	Yes	No	Yes/No
Distribution	Vdss (human) (log L/kg)	2.25	0.99	0.34	0.74	1.15	−0.01	−0.84	−0.83	0.20	0.82	−0.21	−0.10	0.63	Low: < −0.15 High: > 0.45
	Fraction unbound (human) (Fu)	0.26	0.10	0.08	0.11	0.16	0.06	0.162	0.47	0.61	0.14	0.003	0.06	0.54	—
	BBB permeability	−1.86	−1.71	−0.19	−0.71	−0.90	−0.57	0.162	−0.64	0.62	−0.73	−0.19	−0.37	0.51	Good: > 0.3 Poor: < −1
	CNS permeability	−4.60	−4.80	−2.15	−2.97	−2.25	−2.21	−4.14	−2.95	0.62	−2.06	−3.02	−3.16	−2.25	Permeable: > −2 Not permeable: < −3
Metabolism	CYP2D6 substrate	No	No	No	No	No	No	No	No	No	No	No	No	No	Yes/No
	CYP3A4 substrate	No	No	Yes	No	No	No	No	No	No	No	Yes	Yes	No	Yes/No
	CYP1A2 inhibitor	No	No	Yes	No	Yes	Yes	No	No	No	Yes	No	No	No	Yes/No
	CYP2C19 inhibitor	No	No	Yes	No	No	No	No	No	No	Yes	Yes	Yes	No	Yes/No
	CYP2C9 inhibitor	No	No	Yes	No	Yes	No	No	No	No	No	Yes	No	No	Yes/No
	CYP2D6 inhibitor	No	No	No	No	No	No	No	No	No	No	No	No	Yes	Yes/No
	CYP3A4 inhibitor	No	No	Yes	No	No	No	No	No	No	No	Yes	Yes	No	Yes/No
Excretion	Total clearance (log mL/min/kg)	0.47	0.21	0.66	0.04	0.49	0.06	−0.07	1.28	1	0.56	0.07	0.09	0.56	Higher is better
	Renal OCT2 substrate	No	No	No	No	No	No	No	No	No	No	No	No	No	Yes/No
Toxicity	AMES toxicity	No	No	No	No	No	No	No	No	No	No	No	No	No	Yes/No
	hERG I inhibitor	No	No	No	No	No	No	No	No	No	No	No	No	No	Yes/No
	Max. tolerated dose (human) (log mg/kg/day)	0.44	0.52	0.09	0.25	0.49	−0.17	0.40	0.32	0.68	0.33	−0.22	0.01	0.38	—
	Oral rat acute toxicity (LD_50_) (mol/kg)	2.52	2.50	2.22	2.04	2.45	1.79	2.80	1.86	2.04	2.45	2.17	2.13	3.40	—
	Hepatotoxicity	No	No	No	No	No	No	No	No	No	No	No	No	No	Yes/No

Bioavailability radar results are summarized in Figure S5. Overall, the results indicate that our best leads, being physiochemically polar in nature, exhibit poor oral bioavailability compared to the standard rivastigmine. Therefore, these leads may require specialized formulation strategies such as prodrug design by masking polar groups with lipophilic moieties or incorporation into lipid‐based nanocarrier systems (Xiao et al. 2022). Then, ADMET prediction indicated promising pharmacokinetic profiles for all compounds, comparable to rivastigmine. Notably, acteoside showed a more favorable distribution profile than rivastigmine (Vdss = 2.255 log L/kg vs. 0.625 log L/kg, Fu = 0.26 vs. 0.54). Also, acteoside exhibited a more favorable distribution profile than rivastigmine (Vdss = 2.255 log L/kg vs. 0.625 log L/kg, Fu = 0.26 vs. 0.54). Despite the fact that rivastigmine had the highest BBB permeability score (0.51), both acacetin and luteolin also showed potential as CNS drug candidates with scores of −2.15 and −2.25, respectively, which were like rivastigmine's score of −2.25.

**FIGURE 6:**
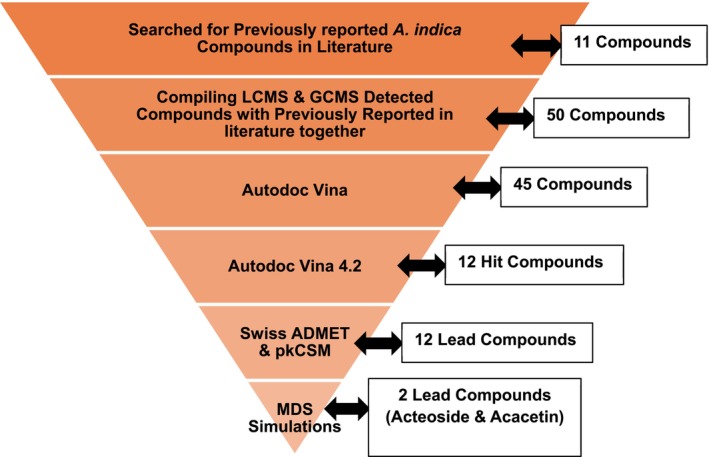
Overview of the screening process for the generation of three prime leads acteoside and acacetin.

Metabolic evaluation yielded varied results. Acteoside displayed no cytochrome P450 inhibition, whereas acacetin inhibited all isoforms except CYP2D6, and luteolin inhibited CYP1A2 and CYP2C9. Regarding excretion, the three best leads (acteoside, acacetin, and luteolin) showed comparable or higher total clearance (log mL/min/kg) compared to rivastigmine. Additionally, all compounds were negative for renal OCT2 substrate, a crucial parameter for oral formulations. Toxicity analysis revealed a non‐hepatotoxic, non‐mutagenic (AMES test), and negative hERG I inhibitor profile for all hits, like rivastigmine. However, acteoside and luteolin exhibited the highest predicted human maximum tolerated dose (0.44 and 0.49 log mg/kg/day) compared to rivastigmine (0.382 log mg/kg/day). The oral rat acute toxicity (LD_50_) for these three best leads was also comparable to rivastigmine. Overall, based on favorable ADMET profiles, potential AChE inhibition, and additional factors (acteoside's past association with AD and abundance of acacetin/luteolin in the extract), acteoside, acacetin, and luteolin were identified as the most promising lead compounds for further development as AChE inhibitors for Alzheimer's disease. In conclusion, Figure 6 schematically depicts the entire process of prime lead selection, encompassing the initial identification of compounds, followed by hit generation, and culminating in the evaluation of drug‐likeness and ADMET properties.

### 3.6 Molecular Dynamics Simulations

This study employed a 100 ns MD simulation to investigate the binding stability of complexes formed between lead compounds (acteoside and acacetin) and AChE. Root Mean Square Deviation (RMSD) analysis served as the primary metric to assess complex stability. Lower RMSD values correspond to a more stable complex with minimal fluctuations, whereas higher values indicate potential structural instability (Bhattacharya et al. 2025).

**FIGURE 7:**
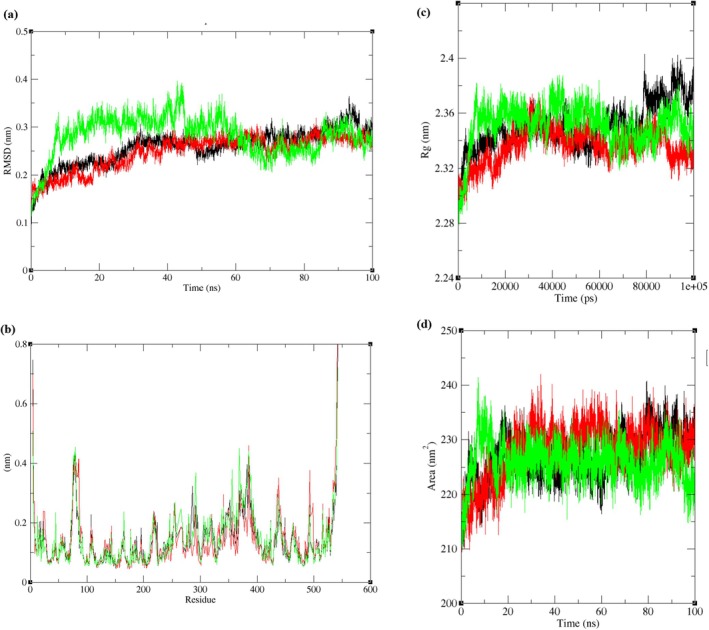
Molecular dynamics features of acteoside and acacetin for acetylcholinesterase during 100 ns of simulation (a) RMSD (b) RMSF (c) Rg (d) SASA. Color indicators: black (apo‐enzyme), red (acteoside), and green (acacetin).

The RMSD analysis indicated that the alpha carbon of apo AChE remained within a range of 0.1–0.35 nm throughout the simulation (Figure 7a). Notably, the acteoside‐AChE complex demonstrated remarkable stability, with consistently lower RMSD values between 0.1 and 0.3 nm. Initially, the RMSD values for the acacetin‐AChE complex ranged from 0.1 to 0.4 nm. However, as the simulation progressed, this complex also exhibited enhanced stability, with RMSD values decreasing to a range of 0.2–0.3 nm. Furthermore, RMSF analysis was conducted to evaluate the flexibility and stability of the complexes by examining atomic deviations within residues. Lower RMSF values indicate greater stability and less flexibility (Bhattacharya et al. 2025). The RMSF profile (Figure 7b) showed minimal fluctuations for both acteoside and acacetin, suggesting well‐defined and stable structures for the complexes.

The radius of gyration (Rg) was used as another metric to assess the compactness and rigidity of the protein‐ligand complexes. Lower Rg values signify a more compact and rigid complex structure due to favorable protein‐ligand interactions (Rehman et al. 2022). The Rg analysis for both the acteoside‐AChE and acacetin‐AChE complexes (Figure 7c) supported this observation. The Rg values were relatively lower and constant, ranging between 2.28 and 2.38 nm, compared to the alpha carbon of apo AChE, which ranged between 2.28 and 2.40 nm. This suggests that the lead‐induced complexes adopted more compact and rigid conformations. Solvent accessible surface area (SASA) analysis provided insights into potential conformational changes experienced by the protein and ligand during binding. An increase in SASA indicates possible alterations in protein or ligand orientation, whereas a decrease suggests that the ligand induces a more compact conformation for the protein upon binding. The SASA profile for both leads showed a decreasing trend compared to the alpha carbon of apo‐AChE, suggesting potential conformational changes (Figure 7d).

**FIGURE 8:**
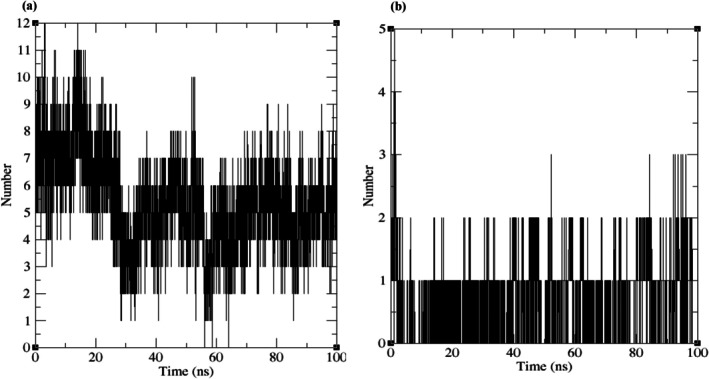
Number of hydrogen bond interactions between (a) acetylcholinesterase and acteoside, (b) acetylcholinesterase and acacetin during 100 ns simulation.

Finally, the number of intermolecular hydrogen bonds was monitored to assess the binding affinity between the complexes formed by acteoside and acacetin with AChE. The acteoside complex initially formed 4–12 hydrogen bonds, followed by a gradual stabilization at 2–8 bonds, indicating a favorable interaction (Figure 8a). The acacetin‐AChE complex also formed 0–2 hydrogen bonds throughout the simulation (Figure 8b), indicating stable interactions with the target apo AChE.

## 4 Discussion

Alzheimer's disease (AD) remains a significant public health burden, characterized by progressive decline in memory and cognitive function. A hallmark of AD is a deficiency in acetylcholine (ACh), a crucial neurotransmitter for cognition. AD pathology involves the buildup of neurofibrillary tangles (NFTs) and senile plaques (SPs) in the brain (Kumar et al. 2015; Silvestrelli et al. 2006), leading to chronic inflammation and increased activity of the enzyme AChE in affected areas. This ultimately results in ACh deficiency (Dos Santos Picanco et al. 2018). Another key mechanism implicated in AD development is sustained oxidative stress and neuroinflammation (Agostinho et al. 2010; Verdile et al. 2015). Its activation worsens AD by promoting amyloid plaque formation and tau hyperphosphorylation (NFT development) (Agostinho et al. 2010; Kinney et al. 2018).

Existing research demonstrates the effectiveness of phenolic compounds in hindering AChE activity, which translates to a prolonged presence of ACh in the synaptic cleft, potentially improving cognitive function in AD (Taslimi et al. 2017; Zaidi et al. 2019). These compounds achieve AChE inhibition through various mechanisms including competitive and non‐competitive inhibition. Phenolic compounds from medicinal plants have garnered growing interest for their ability to inhibit AChE activity (Shiao et al. 2017). Therefore, our study aimed to investigate the potential of *A. indica* derived phenolic compounds to inhibit AChE activity and their possible mechanisms in AD management.

To elucidate the possible mechanisms of *A. indica* derived phenolic compounds for AD management, three lead phenolic compounds (acteoside, acacetin, and luteolin) were selected from AiME based on a combined approach considering molecular docking scores, drug‐likeness, ADMET parameters, and their reported associations with amyloid plaque reduction (Nisa et al. 2023; Park et al. 2018; Shiao et al. 2017). Our selection process identified acteoside as one of the most promising leads. Notably, previous research has demonstrated its ability to inhibit β‐amyloid peptide and the NF‐kB pathway (He et al. 2011; Li et al. 2021; Shiao et al. 2017; Xiao et al. 2022), which aligns with our hypothesized mechanism for *A. indica'*s impact on AD. Furthermore, LCMS/MS analysis confirmed that acacetin and luteolin, the other two selected leads, were abundantly present in the AiME.

**FIGURE 9:**
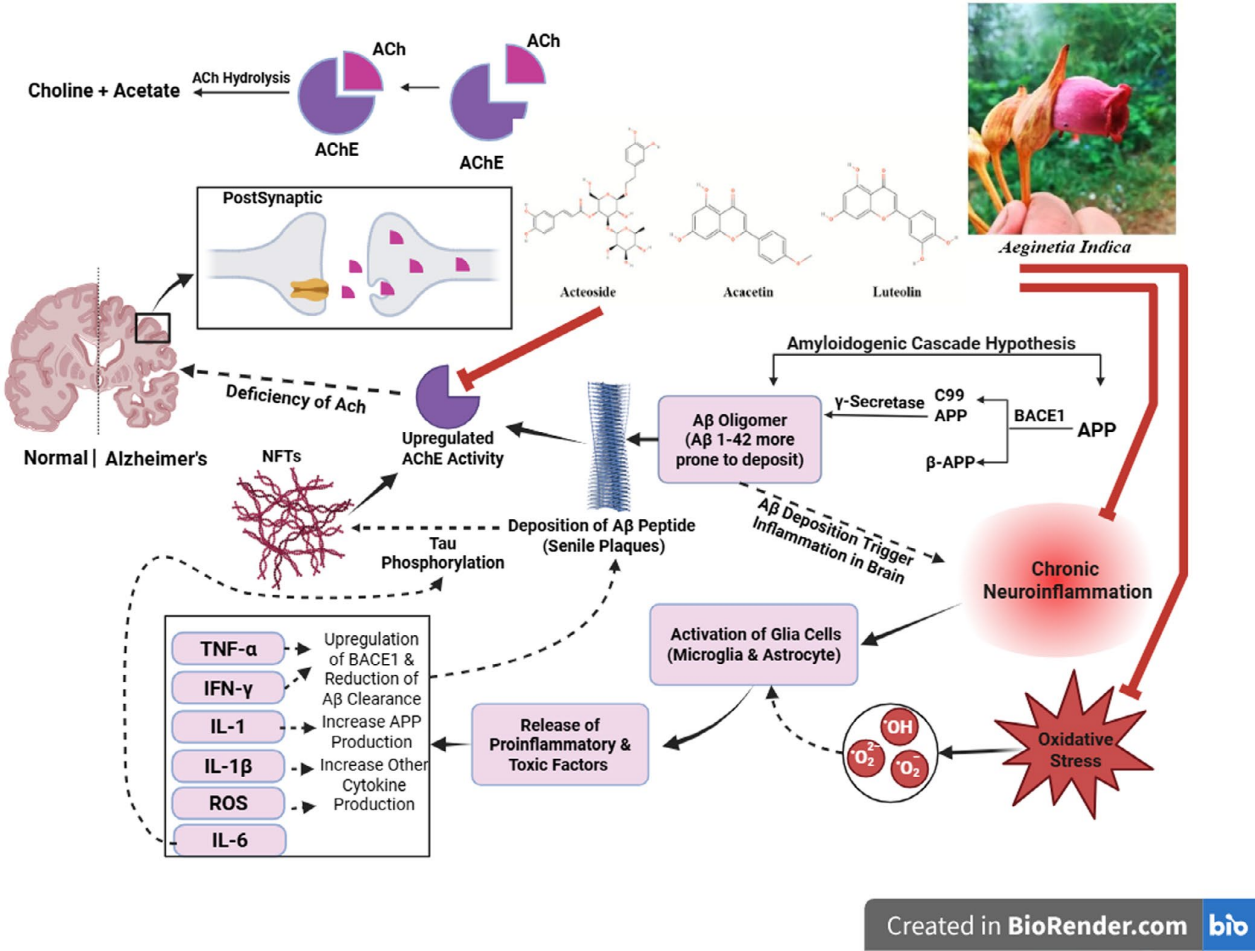
The possible mechanistic pathway of *Aeginetia indica* in AD management.

Molecular docking simulations provided further support for these selections. The study revealed strong hydrogen bond formation between the selected lead compounds (acteoside, acacetin, and luteolin) and the catalytic site of AChE. The findings suggest their potential to competitively inhibit AChE, potentially altering AD progression. Beyond AChE inhibition, our study also revealed significant anti‐inflammatory and antioxidant properties of *A. indica* . These properties could offer additional benefits by mitigating a third core pathophysiological aspect of AD progression. Furthermore, MD simulations demonstrated stable and rigid interactions between the two most promising leads (acteoside and acacetin) and AChE, further strengthening their potential as AChE inhibitors. To show the possible pathways through which *A. indica* may target oxidative stress, neuroinflammation, and AChE activity in AD, a mechanistic pathway is provided in Figure 9.

In a nutshell, this study, together with previously reported pharmacological activities of *A. indica* (including anti‐inflammatory, antipyretic, analgesic, hepatoprotective, and anti‐diabetic effects) (Bhuiyan et al. 2025; Hong et al. 2015; Lin et al. 2018; Parnell 2001; Reza et al. 2021, 2020), highlights its potential as a promising candidate for nutraceutical development. Positioning *A. indica* within the framework of food and nutritional sciences opens new avenues for formulating plant‐based supplements that can serve as natural alternatives to conventional therapeutics. Such nutraceuticals may provide multifunctional health benefits for managing everyday health challenges while minimizing the adverse side effects often associated with synthetic drugs. This perspective underscores the importance of integrating medicinal plants into functional food research, thereby bridging traditional pharmacology with modern nutritional science.

## 5 Conclusion

The present study investigated the antioxidant, anti‐inflammatory, and acetylcholinesterase inhibitory properties of phenolic phytoconstituents from AiME. A multifaceted approach, including in vitro antioxidant and acetylcholinesterase assays, validated the efficacy of AiME as both an antioxidant and acetylcholinesterase inhibitor (AChEI). In vivo anti‐inflammatory tests showed significant activity, whereas molecular docking studies supported the effective binding of acteoside, acacetin, and luteolin to the AChE catalytic site, suggesting a mechanistic basis for the observed AChE inhibition. Molecular dynamics simulations demonstrated favorable rigidity and stability of acteoside and acacetin with AChE during 100 ns simulations. Although these findings highlight AiME as a promising natural source of bioactive compounds that may influence multiple neurodegenerative pathways involved in AD pathogenesis. Further AD relevant investigations, including evaluation of amyloid and tau‐related markers as well as extensive clinical studies, are needed to establish the effectiveness of AiME as a natural therapeutic candidate for AD. Furthermore, the diverse pharmacological activities of *A. indica* present this herb as a promising candidate for nutraceutical formulations or dietary supplements that may provide multifunctional health benefits in managing everyday health challenges.

## Author Contributions

**Mustafa Abdullah Yilmaz:** data curation, software, validation, formal analysis. **Md. Sharif Reza:** conceptualization, methodology, investigation, writing – original draft. **Nuraniye Eruygur:** conceptualization, supervision, writing – review and editing. **Syed Mumtahin Mannan Siam:** formal analysis, visualization. **Marjanur Rahman Bhuiyan:** conceptualization, investigation, methodology, writing – original draft. **Abbas Tarhan:** validation, formal analysis. **Sadikur Rahman Shuvo:** methodology, software, formal analysis, visualization. **A. F. M. Shahid Ud Daula:** conceptualization, writing – original draft, writing – review and editing, investigation, supervision, resources. **Md. Nazmul Islam Durjoy:** conceptualization, methodology, investigation, writing – original draft. **Md. Shadman Sakib:** formal analysis, visualization. **Oguz Cakir:** data curation, validation.

## Funding

The authors have nothing to report.

## Conflicts of Interest

The authors declare no conflicts of interest.

## Supporting information


**Figure S1:** Mice paw size. (a) Non‐inflamed paw; (b) inflamed paw after carrageenan injection.
**Figure S2:** LC–MS/MS chromatogram of standard compounds.
**Figure S3:** 2d conformations of acetylcholinesterase enzyme (AChE) with five potential ligands: (A) acteoside, (B) acacetin, (C) balanophonin, (D) hesperetin, (E) luteolin, (F) naringenin, (G) ficusal, and (H) rivastigmine (standard) produced by Ligplot+.
**Figure S4:** Possible 3d interactions of (A) balanophonin, (B) hesperetin, (C) naringenin, (D) ficusal with acetylcholinesterase (AchE) (pdb id: 4ey7) (pose predicted by PyMoL).
**Figure S5:** Bioavailability radar of lead compounds of *Aeginetia indica* with standard rivastigmine as following:(A) acteoside, (B) acacetin, (C) balanophonin, (D) hesperetin, (E) luteolin, (F) naringenin, (G) ficusal, and (H) rivastigmine.
**Table S1:** Analytical method validation parameters that belong to the LC–MS/MS method.
**Table S2:** Lists of previously identified components from *Aeginetia indica* for their different bioactivities.
**Table S3:** Bond analysis of the selected phytoconstituents and rivastigmine (standard) with acetylcholinesterase enzyme (AChE).

## Data Availability

The data that support the findings of this study are available from the corresponding author upon reasonable request.

## References

[fsn371886-bib-0001] Agostinho, P. , R. A. Cunha , and C. Oliveira . 2010. “Neuroinflammation, Oxidative Stress and the Pathogenesis of Alzheimer's Disease.” Current Pharmaceutical Design 16, no. 25: 2766–2778. 10.2174/138161210793176572. 20698820

[fsn371886-bib-0002] Ahmed, S. , K. S. Ahmed , M. N. Rahman , et al. 2024. “Polyphenols and Extracts From *Zingiber roseum* (Roxb.) Roscoe Leaf Mitigate Pain, Inflammation and Pyrexia by Inhibiting Cyclooxygenase‐2: An In Vivo and In Silico Studies.” Frontiers in Pharmacology 15: 1344123. 10.3389/fphar.2024.1344123. 38420193 PMC10900100

[fsn371886-bib-0003] Alhakmani, F. , S. Kumar , and S. A. Khan . 2013. “Estimation of Total Phenolic Content, In Vitro Antioxidant and Anti‐Inflammatory Activity of Flowers of *Moringa oleifera* .” Asian Pacific Journal of Tropical Biomedicine 3, no. 8: 623–627; discussion 626–627. 10.1016/S2221-1691(13)60126-4. 23905019 PMC3703555

[fsn371886-bib-0004] Alzheimers Association . 2023. “2023 Alzheimer's Disease Facts and Figures.” Alzheimer's & Dementia: The Journal of the Alzheimer's Association 19, no. 4: 1598–1695. 10.1002/alz.13016. 36918389

[fsn371886-bib-0005] Anandjiwala, S. , M. Bagul , M. Parabia , and M. Rajani . 2008. “Evaluation of Free Radical Scavenging Activity of an Ayurvedic Formulation, Panchvalkala.” Indian Journal of Pharmaceutical Sciences 70, no. 1: 31–35. 10.4103/0250-474X.40328. 20390077 PMC2852057

[fsn371886-bib-0006] Anu, O. S. , S. Ahmed , M. T. Islam , V. K. Shah , M. I. Ahmed , and M. A. Rahman . 2025. “Comparative Investigation of Free Radical Scavenging and Cyclic Voltammetric Analyses to Evaluate the Antioxidant Potential of Selective Green Vegetables Extracts.” Scientific Reports 15, no. 1: 31919. 10.1038/s41598-025-12731-y. 40883357 PMC12397375

[fsn371886-bib-0007] Benzie, I. F. , and J. J. Strain . 1996. “The Ferric Reducing Ability of Plasma (FRAP) as a Measure of ‘Antioxidant Power’: The FRAP Assay.” Analytical Biochemistry 239, no. 1: 70–76. 10.1006/abio.1996.0292. 8660627

[fsn371886-bib-0009] Bhattacharya, K. , A. Bhattacharjee , and M. Chakraborty . 2025. “Assessing the Potential of *Psidium guajava* Derived Phytoconstituents as Anticholinesterase Inhibitor to Combat Alzheimer's Disease: An In‐Silico and In Vitro Approach.” Journal of Biomolecular Structure & Dynamics 43, no. 8: 4240–4257. 10.1080/07391102.2024.2301930. 38205777

[fsn371886-bib-0010] Bhuiyan, M. R. , K. S. Ahmed , M. S. Reza , et al. 2025. “Prediction of Angiogenesis Suppression by Myricetin From *Aeginetia indica* via Inhibiting VEGFR2 Signaling Pathway Using Computer‐Aided Analysis.” Heliyon 11, no. 2: e41749. 10.1016/j.heliyon.2025.e41749. 39897831 PMC11786634

[fsn371886-bib-0011] Birks, J. S. , and J. Grimley Evans . 2015. “Rivastigmine for Alzheimer's Disease.” Cochrane Database of Systematic Reviews 4: CD001191. 10.1002/14651858.CD001191.pub3. 25858345

[fsn371886-bib-0013] Daina, A. , O. Michielin , and V. Zoete . 2017. “SwissADME: A Free Web Tool to Evaluate Pharmacokinetics, Drug‐Likeness and Medicinal Chemistry Friendliness of Small Molecules.” Scientific Reports 7, no. 1: 42717. 10.1038/srep42717. 28256516 PMC5335600

[fsn371886-bib-0014] de Araújo, L. P. , E. N. Silva , P. P. Corsetti , C. A. Tagliati , N. J. F. da Silveira , and L. A. de Almeida . 2025. “Virtual Screening and Bioisosterism of Natural Products for Targeting A2Ar, BTK, P38‐MAPK, PAD‐4, and TNF‐α in Psoriatic Symptomatology Modulation.” BMC Methods 2, no. 1: 20. 10.1186/s44330-025-00036-5.

[fsn371886-bib-0015] Diment, D. , O. Musl , M. Balakshin , and D. Rigo . 2025. “Guidelines for Evaluating the Antioxidant Activity of Lignin via the 2,2‐Diphenyl‐1‐Picrylhydrazyl (DPPH) Assay.” ChemSusChem 18, no. 10: e202402383. 10.1002/cssc.202402383. 40105287

[fsn371886-bib-0016] Dos Santos Picanco, L. C. , P. F. Ozela , M. de Fatima de Brito Brito , et al. 2018. “Alzheimer's Disease: A Review From the Pathophysiology to Diagnosis, New Perspectives for Pharmacological Treatment.” Current Medicinal Chemistry 25, no. 26: 3141–3159. 10.2174/0929867323666161213101126. 30191777

[fsn371886-bib-0017] Ellman, G. L. , K. D. Courtney , V. Andres , and R. M. Featherstone . 1961. “A New and Rapid Colorimetric Determination of Acetylcholinesterase Activity.” Biochemical Pharmacology 7, no. 2: 88–95. 10.1016/0006-2952(61)90145-9. 13726518

[fsn371886-bib-0019] Gandla, K. , F. Islam , M. Zehravi , et al. 2023. “Natural Polymers as Potential P‐Glycoprotein Inhibitors: Pre‐ADMET Profile and Computational Analysis as a Proof of Concept to Fight Multidrug Resistance in Cancer.” Heliyon 9, no. 9: e19454. 10.1016/j.heliyon.2023.e19454. 37662819 PMC10472248

[fsn371886-bib-0020] Gulcin, İ. , and S. H. Alwasel . 2025. “Fe^3+^ Reducing Power as the Most Common Assay for Understanding the Biological Functions of Antioxidants.” Processes 13, no. 5: 1296. 10.3390/pr13051296.

[fsn371886-bib-0021] He, J. , X.‐P. Hu , Y. Zeng , et al. 2011. “Advanced Research on Acteoside for Chemistry and Bioactivities.” Journal of Asian Natural Products Research 13, no. 5: 449–464. 10.1080/10286020.2011.568940. 21534045

[fsn371886-bib-0022] Ho, C.‐T. 1992. “Phenolic Compounds in Food.” In ACS Symposium Series: Vol. 507. Phenolic Compounds in Food and Their Effects on Health II, vol. 507, 2–7. American Chemical Society. 10.1021/bk-1992-0507.ch001.

[fsn371886-bib-0023] Ho, J.‐C. , C.‐M. Chen , Z.‐Q. Li , and L.‐C. Row . 2004. “Phenylpropanoid Glycosides From the Parasitic Plant, *Aeginetia indica* .” Journal of the Chinese Chemical Society 51, no. 5A: 1073–1076. 10.1002/jccs.200400160.

[fsn371886-bib-0024] Ho, J.‐C. , C.‐M. Chen , and L.‐C. Row . 2003. “Neolignans From the Parasitic Plants. Part 1. *Aeginetia indica* .” Journal of the Chinese Chemical Society 50, no. 6: 1271–1274. 10.1002/jccs.200300183.

[fsn371886-bib-0025] Hong, L. , Z. Guo , K. Huang , et al. 2015. “Ethnobotanical Study on Medicinal Plants Used by Maonan People in China.” Journal of Ethnobiology and Ethnomedicine 11, no. 1: 32. 10.1186/s13002-015-0019-1. 25925830 PMC4449599

[fsn371886-bib-0026] Irshad, A. , R. Jawad , Q. Mushtaq , A. Spalletta , P. Martin , and U. Ishtiaq . 2025. “Determination of Antibacterial and Antioxidant Potential of Organic Crude Extracts From *Malus domestica* , *Cinnamomum verum* and *Trachyspermum ammi* .” Scientific Reports 15, no. 1: 976. 10.1038/s41598-024-83506-0. 39762362 PMC11704246

[fsn371886-bib-0027] Jain, M. , R. Dhariwal , K. Bhardava , et al. 2024. “In Silico and In Vitro Profiling of Curcumin and Its Derivatives as a Potent Acetylcholinesterase Inhibitor.” Biocatalysis and Agricultural Biotechnology 56: 103022. 10.1016/j.bcab.2024.103022.

[fsn371886-bib-0028] Jiang, Y. , and H. Gao . 2018. “Pharmacophore‐Based Drug Design for Potential AChE Inhibitors From Traditional Chinese Medicine Database.” Bioorganic Chemistry 76: 400–414. 10.1016/j.bioorg.2017.12.015. 29258018

[fsn371886-bib-0029] Kinney, J. W. , S. M. Bemiller , A. S. Murtishaw , A. M. Leisgang , A. M. Salazar , and B. T. Lamb . 2018. “Inflammation as a Central Mechanism in Alzheimer's Disease.” Alzheimer's & Dementia (NY) 4: 575–590. 10.1016/j.trci.2018.06.014. PMC6214864 30406177

[fsn371886-bib-0030] Kumar, A. , A. Singh , and Ekavali . 2015. “A Review on Alzheimer's Disease Pathophysiology and Its Management: An Update.” Pharmacological Reports 67, no. 2: 195–203. 10.1016/j.pharep.2014.09.004. 25712639

[fsn371886-bib-0031] Kumar, H. , A. K. Datusalia , and G. L. Khatik . 2024. “Virtual Screening of Acetylcholinesterase Inhibitors Through Pharmacophore‐Based 3D‐QSAR Modeling, ADMET, Molecular Docking, and MD Simulation Studies.” In Silico Pharmacology 12, no. 1: 13. 10.1007/s40203-024-00189-1. 38370859 PMC10873251

[fsn371886-bib-0032] Kurisu, M. , Y. Miyamae , K. Murakami , et al. 2013. “Inhibition of Amyloid β Aggregation by Acteoside, a Phenylethanoid Glycoside.” Bioscience, Biotechnology, and Biochemistry 77, no. 6: 1329–1332. 23748773 10.1271/bbb.130101

[fsn371886-bib-0033] Li, X. , X. Feng , X. Sun , N. Hou , F. Han , and Y. Liu . 2022. “Global, Regional, and National Burden of Alzheimer's Disease and Other Dementias, 1990–2019.” Frontiers in Aging Neuroscience 14: 937486. 10.3389/fnagi.2022.937486. 36299608 PMC9588915

[fsn371886-bib-0034] Li, Y.‐Q. , Y. Chen , S.‐Q. Jiang , et al. 2021. “An Inhibitor of NF‐κB and an Agonist of AMPK: Network Prediction and Multi‐Omics Integration to Derive Signaling Pathways for Acteoside Against Alzheimer's Disease.” Frontiers in Cell and Developmental Biology 9: 652310. 10.3389/fcell.2021.652310. 34350171 PMC8327963

[fsn371886-bib-0035] Lin, C.‐W. , C.‐W. Lo , C.‐N. Tsai , T.‐C. Pan , P.‐Y. Chen , and M.‐J. Yu . 2018. “ *Aeginetia indica* Decoction Inhibits Hepatitis C Virus Life Cycle.” International Journal of Molecular Sciences 19, no. 1: 208. 10.3390/ijms19010208. 29315273 PMC5796157

[fsn371886-bib-0036] Liu, Y. H. , M. L. Li , M. Y. Hsu , et al. 2012. “Effects of a Chinese Herbal Medicine, Guan‐Jen‐Huang ( *Aeginetia indica* Linn.), on Renal Cancer Cell Growth and Metastasis.” Evidence‐Based Complementary and Alternative Medicine 2012: 935860. 10.1155/2012/935860. 22028734 PMC3199064

[fsn371886-bib-0038] Martínez‐Zamora, L. , M. Cano‐Lamadrid , F. Artés‐Hernández , et al. 2023. “Flavonoid Extracts From Lemon By‐Products as a Functional Ingredient for New Foods: A Systematic Review.” Food 12, no. 19: 3687. 10.3390/foods12193687. PMC10573073 37835340

[fsn371886-bib-0039] Mazumder, T. , T. Hasan , K. S. Ahmed , et al. 2022. “Phenolic Compounds and Extracts From *Crotalaria calycina* Schrank Potentially Alleviate Pain and Inflammation Through Inhibition of Cyclooxygenase‐2: An In Vivo and Molecular Dynamics Studies.” Heliyon 8, no. 12: e12368. 10.1016/j.heliyon.2022.e12368. 36590510 PMC9800535

[fsn371886-bib-0040] Nisa, N. , B. Rasmita , C. Arati , et al. 2023. “Repurposing of Phyto‐Ligand Molecules From the Honey Bee Products for Alzheimer's Disease as Novel Inhibitors of BACE‐1: Small Molecule Bioinformatics Strategies as Amyloid‐Based Therapy.” Environmental Science and Pollution Research 30, no. 17: 51143–51169. 10.1007/s11356-023-25943-4. 36808033

[fsn371886-bib-0041] On‐Nom, N. , S. Thangsiri , W. Inthachat , et al. 2024. “Optimized Conditions for the Extraction of Phenolic Compounds From *Aeginetia indica* L. and Its Potential Biological Applications.” Molecules 29, no. 5: 1050. 10.3390/molecules29051050. 38474563 PMC10935255

[fsn371886-bib-0043] Park, S. , D. S. Kim , S. Kang , and H. J. Kim . 2018. “The Combination of Luteolin and l‐Theanine Improved Alzheimer Disease–Like Symptoms by Potentiating Hippocampal Insulin Signaling and Decreasing Neuroinflammation and Norepinephrine Degradation in Amyloid‐β–Infused Rats.” Nutrition Research 60: 116–131. 10.1016/j.nutres.2018.09.010. 30527255

[fsn371886-bib-0044] Parnell, J. 2001. “A Revision of Orobanchaceae in Thailand.” Thai Forest Bulletin (Botany) 29: 72–80.

[fsn371886-bib-0045] Parveen, B. , V. Rajinikanth , and M. Narayanan . 2025. “Natural Plant Antioxidants for Food Preservation and Emerging Trends in Nutraceutical Applications.” Discover Applied Sciences 7, no. 8: 845. 10.1007/s42452-025-07464-6.

[fsn371886-bib-0047] Percie du Sert, N. , A. Ahluwalia , S. Alam , et al. 2020. “Reporting Animal Research: Explanation and Elaboration for the ARRIVE Guidelines 2.0.” PLoS Biology 18, no. 7: e3000411. 10.1371/journal.pbio.3000411. 32663221 PMC7360025

[fsn371886-bib-0048] Rehman, S. , U. Ali Ashfaq , M. Sufyan , I. Shahid , B. Ijaz , and M. Hussain . 2022. “The Insight of In Silico and In Vitro Evaluation of *Beta vulgaris* Phytochemicals Against Alzheimer's Disease Targeting Acetylcholinesterase.” PLoS One 17, no. 3: e0264074. 10.1371/journal.pone.0264074. 35239683 PMC8893657

[fsn371886-bib-0049] Reza, M. S. , M. Jashimuddin , J. Ahmed , et al. 2021. “Pharmacological Investigation of Analgesic and Antipyretic Activities of Methanol Extract of the Whole Part of *Aeginetia indica* .” Journal of Ethnopharmacology 271: 113915. 10.1016/j.jep.2021.113915. 33567308

[fsn371886-bib-0050] Reza, M. S. , M. S. R. Shuvo , M. M. Hassan , et al. 2020. “Antidiabetic and Hepatoprotective Potential of Whole Plant Extract and Isolated Compounds of *Aeginetia indica* .” Biomedicine & Pharmacotherapy 132: 110942. 10.1016/j.biopha.2020.110942. 33254438

[fsn371886-bib-0051] Roca, C. , C. Requena , V. Sebastian‐Perez , et al. 2018. “Identification of New Allosteric Sites and Modulators of AChE Through Computational and Experimental Tools.” Journal of Enzyme Inhibition and Medicinal Chemistry 33, no. 1: 1034–1047. 10.1080/14756366.2018.1476502. 29873262 PMC6010107

[fsn371886-bib-0054] Sapkota, P. P. 2014. “Religious Culture and Medicinal Plants: An Anthropological Study.” Dhaulagiri Journal of Sociology and Anthropology 7: 197–224. 10.3126/dsaj.v7i0.10443.

[fsn371886-bib-0055] Scheltens, P. , B. De Strooper , M. Kivipelto , et al. 2021. “Alzheimer's Disease.” Lancet 397, no. 10284: 1577–1590. 10.1016/S0140-6736(20)32205-4. 33667416 PMC8354300

[fsn371886-bib-0056] Settu, R. , D. Selvaraj , and I. A. Padikasan . 2021. “GCMS Profiling and In Silico Screening of Alpha‐Amylase Inhibitors in Traditional Pigmented Rice Varieties ( *Oryza sativa* Linn) of Tamil Nadu.” Food Bioscience 42: 101154. 10.1016/j.fbio.2021.101154.

[fsn371886-bib-0057] Shiao, Y.‐J. , M.‐H. Su , H.‐C. Lin , and C.‐R. Wu . 2017. “Acteoside and Isoacteoside Protect Amyloid β Peptide Induced Cytotoxicity, Cognitive Deficit and Neurochemical Disturbances In Vitro and In Vivo.” International Journal of Molecular Sciences 18, no. 4: 895. 28441758 10.3390/ijms18040895 PMC5412474

[fsn371886-bib-0058] Silvestrelli, G. , A. Lanari , L. Parnetti , D. Tomassoni , and F. Amenta . 2006. “Treatment of Alzheimer's Disease: From Pharmacology to a Better Understanding of Disease Pathophysiology.” Mechanisms of Ageing and Development 127, no. 2: 148–157. 10.1016/j.mad.2005.09.018. 16278007

[fsn371886-bib-0059] Sun, S. , Z. Liu , M. Lin , N. Gao , and X. Wang . 2024. “Polyphenols in Health and Food Processing: Antibacterial, Anti‐Inflammatory, and Antioxidant Insights.” Frontiers in Nutrition 11: 1456730. 10.3389/fnut.2024.1456730. 39224187 PMC11366707

[fsn371886-bib-0060] Taslimi, P. , C. Caglayan , and İ. Gulcin . 2017. “The Impact of Some Natural Phenolic Compounds on Carbonic Anhydrase, Acetylcholinesterase, Butyrylcholinesterase, and α‐Glycosidase Enzymes: An Antidiabetic, Anticholinergic, and Antiepileptic Study.” Journal of Biochemical and Molecular Toxicology 31, no. 12: e21995. 10.1002/jbt.21995. 28902458

[fsn371886-bib-0061] Verdile, G. , K. N. Keane , V. F. Cruzat , et al. 2015. “Inflammation and Oxidative Stress: The Molecular Connectivity Between Insulin Resistance, Obesity, and Alzheimer's Disease.” Mediators of Inflammation 2015: 105828. 10.1155/2015/105828. 26693205 PMC4674598

[fsn371886-bib-0062] Xiao, Y. , Q. Ren , and L. Wu . 2022. “The Pharmacokinetic Property and Pharmacological Activity of Acteoside: A Review.” Biomedicine & Pharmacotherapy 153: 113296. 10.1016/j.biopha.2022.113296. 35724511 PMC9212779

[fsn371886-bib-0063] Yang, J. , B. Ju , Y. Yan , et al. 2017. “Neuroprotective Effects of Phenylethanoid Glycosides in an In Vitro Model of Alzheimer's Disease.” Experimental and Therapeutic Medicine 13, no. 5: 2423–2428. 10.3892/etm.2017.4254. 28565858 PMC5443222

[fsn371886-bib-0064] Yilmaz, M. A. 2020. “Simultaneous Quantitative Screening of 53 Phytochemicals in 33 Species of Medicinal and Aromatic Plants: A Detailed, Robust and Comprehensive LC–MS/MS Method Validation.” Industrial Crops and Products 149: 112347. 10.1016/j.indcrop.2020.112347.

[fsn371886-bib-0065] Zaidi, H. , S. Ouchemoukh , N. Amessis‐Ouchemoukh , et al. 2019. “Biological Properties of Phenolic Compound Extracts in Selected Algerian Honeys—The Inhibition of Acetylcholinesterase and α‐Glucosidase Activities.” European Journal of Integrative Medicine 25: 77–84. 10.1016/j.eujim.2018.11.008.

