# Supplementary material for: Potential of Aeginetia indica for Alzheimer's Disease Management: In Vitro, In Vivo, and Computational Insights
Source: Food Sci Nutr. 2026 Jun 19;14(6):e71886. doi: 10.1002/fsn3.71886 (PMC13280805; doi:10.1002/fsn3.71886)
Supplement: Supplementary file 1 — Figure S1: Mice paw size. (a) Non‐inflamed paw; (b) inflamed paw after carrageenan injection. Figure S2: LC–MS/MS chromatogram of standard compounds. Figure S3: 2d conformations of acetylcholinesterase enzyme (AChE) with five potential ligands: (A) acteoside, (B) acacetin, (C) balanophonin, (D) hesperetin, (E) luteolin, (F) naringenin, (G) ficusal, and (H) rivastigmine (standard) produced by Ligplot+. Figure S4: Possible 3d interactions of (A) balanophonin, (B) hesperetin, (C) naringenin, (D) ficusal with acetylcholinesterase (AchE) (pdb id: 4ey7) (pose predicted by PyMoL). Figure S5: Bioavailability radar of lead compounds of Aeginetia indica with standard rivastigmine as following:(A) acteoside, (B) acacetin, (C) balanophonin, (D) hesperetin, (E) luteolin, (F) naringenin, (G) ficusal, and (H) rivastigmine. Table S1: Analytical method validation parameters that belong to the LC–MS/MS method. Table S2: Lists of previously identified components from Aeginetia indica for their different bioactivities. Table S3: Bond analysis of the selected phytoconstituents and rivastigmine (standard) with acetylcholinesterase enzyme (AChE). [file FSN3-14-e71886-s001.docx]

**Supplementary Files**

**S1A. Methodology**

**2.5 LC─MS/MS Analysis**

In the present study, the quantitative evaluation of 53 phytochemicals and 3 deuterated internal standards was conducted using a Shimadzu-Nexera model ultrahigh performance liquid chromatograph (UHPLC) coupled with a tandem mass spectrometer according to the method demonstrated by Yilmaz (2020). The UHPLC system utilized for reversed-phase chromatography consisted of an autosampler (SIL-30AC model), a column oven (CTO-10ASvp model), binary pumps (LC-30AD model), and a degasser (DGU-20A3R model). The chromatographic settings were tuned to provide optimal separation for 53 phytochemicals and mitigate the impacts of suppression. Various columns, including the Agilent Poroshell 120 EC-C18 (150 mm × 2.1 mm, 2.7 µm) and RP-C18 Inertsil ODS-4 (100 mm × 2.1 mm, 2 µm), were tested with different mobile phases such as acetonitrile and methanol. Additionally, mobile phase additives like ammonium formate, formic acid, ammonium acetate, and acetic acid were evaluated. The column temperature varied between 25°C and 40°C to determine the optimal conditions. Ultimately, chromatographic separation was performed using an Agilent Poroshell 120 EC-C18 (150 mm × 2.1 mm, 2.7 µm) analytical column in reversed-phase mode, with the column temperature set at 40°C. The elution gradient consisted of eluent A (water with 5 mM ammonium formate and 0.1% formic acid) and eluent B (methanol with 5 mM ammonium formate and 0.1% formic acid). The gradient profile was as follows: a linear increase from 20% to 100% B over 0-25 minutes, a constant 100% B from 25-35 minutes, and a decrease back to 20% B from 35-45 minutes. The solvent flow rate and injection volume were set at 0.5 mL/min and 5 µL, respectively (Yilmaz, 2020). Prior to LC─MS/MS analysis, the extract was diluted with methanol to achieve a final concentration of 1 mg/mL. The test samples were maintained at a consistent temperature of 15 degrees Celsius throughout the experiment. The analysis of individual phytochemicals in the extracts was conducted using a set of 56 standard phytochemicals. In Supplementary Figure 2 (SF2), the circumstances of the mass spectrometer and chromatograph, the total ion chromatogram, and the analytical validation parameters for the standard compounds are presented for better clarity.

**2.7 In silico Analysis**

This investigation focuses on the *in-silico* docking of a crystal acetylcholinesterase enzyme (AChE) as the target macromolecule. A ligand library of 50 compounds was compiled, incorporating data from Liquid Chromatography with Tandem Mass Spectrometry LCMS/MS, Gas Chromatography-Mass Spectrometry (GCMS), and previously reported literature sources (Ho, Chen, Li, & Row, 2004; Ho, Chen, & Row, 2003). Additionally, the reference drug rivastigmine was included for validation purposes. The docking approach used in this study follows the methodology described in our previous work by (Mazumder et al., 2022), a prior publication by our research group.

**2.7.1 Protein preparation**

The co-crystalized structure of AChE was saved from the RCSB website (https://www.rcsb.org/) in pdb format (PDB ID: 4EY7). Later on, homology modeling was conducted in a trustworthy server (https://swissmodel.expasy.org/) to maintain the universal integrity of the incomplete co-crystalized structure of AChE. A software called PyMoL was used to clean and remove unwanted water and other ligand molecules attached to the co-crystalized structure of AChE. Energy minimization of cleaned protein was carried out by SPDBV tools (version 4.1.0). Afterward, polar and non-polar hydrogens merged with macromolecules by Auto Dock Tools (Version-1.5.6 - The Scripps Research Institute USA). In the end, the final protein was stored in Pdbqt file format for the next step of the study.

**2.7.2 Ligand Preparation**

In order to create a robust ligand library for our in-silico investigation, a combination of approaches was employed. Firstly, compounds identified through LCMS and compounds from GCMS analysis were incorporated together. Secondly, previously reported *A. indica* phytochemicals were obtained from a comprehensive literature review and summarized in supplementary table 2 (ST2) (Jiang & Gao, 2018) (Ho et al., 2004, 2003). To validate the molecular docking study, rivastigmine, a standard drug for AD, was also included in the library. Compounds were downloaded from the PubChem database in sdf file format. PyMol was used to complete the conversion of the format of the downloaded structure from sdf to pdb. Following the addition of polar hydrogen, Kolman charges, and torsion fixing using Auto Dock tools 1.5.6, the ligands were subsequently exported into pdbqt format.

**2.7.3 Molecular docking**

Before conducting docking simulations with AutoDock Vina, the active site of the catalytic enzyme acetylcholinesterase (AChE) (PDB ID: 4EY7), was retrieved from the ligand‑binding pocket of the co‑crystallized protein structure and then also examined using CASTp 3.0 (http://sts.bioe.uic.edu/castp/index.html?4ey7). Tyr72, Trp86, Trp286, Ser293, Phe295, Tyr337, Phe338 and Tyr34 have been identified as crucial key residues in the active site that potentially regulate AChE physiology and activity (Jiang & Gao, 2018; Roca et al., 2018).

To facilitate a detailed analysis of interactions between the active site residue of AChE and potential ligands, a predefined grid box was generated using AutoDock Vina tools 1.5.6. The grid box encompasses the binding site region, specifically centered at coordinates (x, y, z). The AutoGrid position was further optimized to (x: -13.695, y: -43.048, z: 27.319) to ensure optimal ligand conformation during docking. The grid box dimensions were set to (x: 30, y: 30, z: 30) to encompass all the identified active site residues. Additionally, an exhaustiveness setting of 8.0 was employed to enhance the search for the most favorable binding mode.

**2.8 Molecular Dynamics (MD) Simulations**

In this study, 100 ns MD simulations were performed for both acteoside and acacetin with apoenzyme acetylcholinesterase (AChE) using the GROMACS 2021.3 software package. Consistent with our previous laboratory protocols (Ahmed et al., 2024; Mazumder et al., 2022), topology files for the protein and ligand were independently generated using the CHARMM36 force field (CGenFF) server. The AChE-Acteoside complex structure was then constructed by merging these individual topologies. The complex was solvated with a TIP3P water model and neutralized with the addition of counterions. Energy minimization was performed using 5000 steps of steepest descent minimization to eliminate unfavorable initial configurations. To maintain ligand positioning within the complex during the simulation, a position restraint topology was implemented. Subsequently, the system underwent NVT and NPT equilibration steps with temperature coupling applied to the protein-ligand pair for system stabilization. Finally, upon completion of the MD simulations, structural properties including root-mean-square deviation (RMSD), root-mean-square fluctuation (RMSF), radius of gyration (Rg), Solvent Accessible Surface Area (SASA), and the number of hydrogen bonds between the protein and ligand were analyzed.

**References**

Ahmed, S., Ahmed, K. S., Rahman, M. N., Hossain, H., Han, A., Geng, P., … Mamun, A. A. (2024). Polyphenols and extracts from Zingiber roseum (Roxb.) Roscoe leaf mitigate pain, inflammation and pyrexia by inhibiting cyclooxygenase-2: An in vivo and in silico studies. *Frontiers in Pharmacology*, *15*, 1344123. https://doi.org/10.3389/fphar.2024.1344123

Ho, J.-C., Chen, C.-M., Li, Z.-Q., & Row, L.-C. (2004). Phenylpropanoid Glycosides from the Parasitic Plant, Aeginetia Indica. *Journal of the Chinese Chemical Society*, *51*(5A), 1073–1076. https://doi.org/10.1002/jccs.200400160

Ho, J.-C., Chen, C.-M., & Row, L.-C. (2003). Neolignans from the Parasitic Plants. Part 1. Aeginetia Indica. *Journal of the Chinese Chemical Society*, *50*(6), 1271–1274. https://doi.org/10.1002/jccs.200300183

Jiang, Y., & Gao, H. (2018). Pharmacophore-based drug design for potential AChE inhibitors from Traditional Chinese Medicine Database. *Bioorganic Chemistry*, *76*, 400–414. https://doi.org/10.1016/j.bioorg.2017.12.015

Mazumder, T., Hasan, T., Ahmed, K. S., Hossain, H., Debnath, T., Jahan, E., … Daula, A. F. M. S. U. (2022). Phenolic compounds and extracts from *Crotalaria calycina* Schrank potentially alleviate pain and inflammation through inhibition of cyclooxygenase-2: An in vivo and molecular dynamics studies. *Heliyon*, *8*(12), e12368. https://doi.org/10.1016/j.heliyon.2022.e12368

Roca, C., Requena, C., Sebastián-Pérez, V., Malhotra, S., Radoux, C., Pérez, C., … Campillo, N. E. (2018). Identification of new allosteric sites and modulators of AChE through computational and experimental tools. *Journal of Enzyme Inhibition and Medicinal Chemistry*, *33*(1), 1034–1047. https://doi.org/10.1080/14756366.2018.1476502

**S1B. Experimental Results**

**
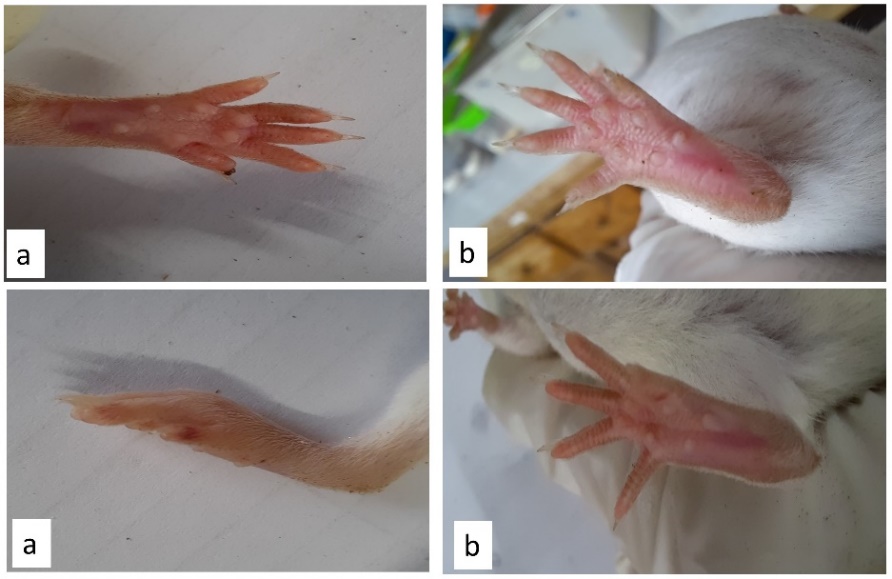
**

**Supplementary Figure 1:** Mice paw size a). Non-inflamed paw; b). Inflamed paw after carrageenan injection


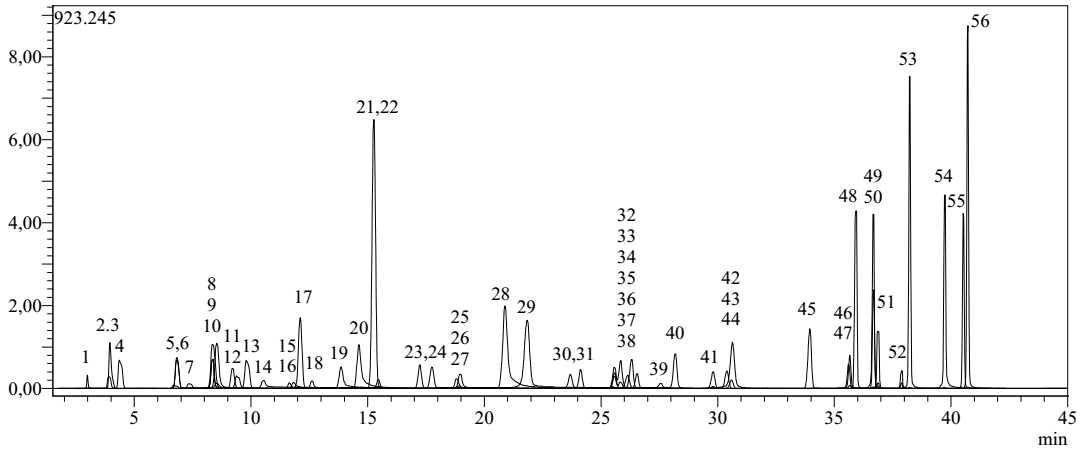


**Supplementary Figure 2:** LC-MS/MS chromatogram of standard compounds.

**(G)**

**(H)**

**(D)**

**(E)**

**(F)**

**(A)**

**(B)**

**(C)**


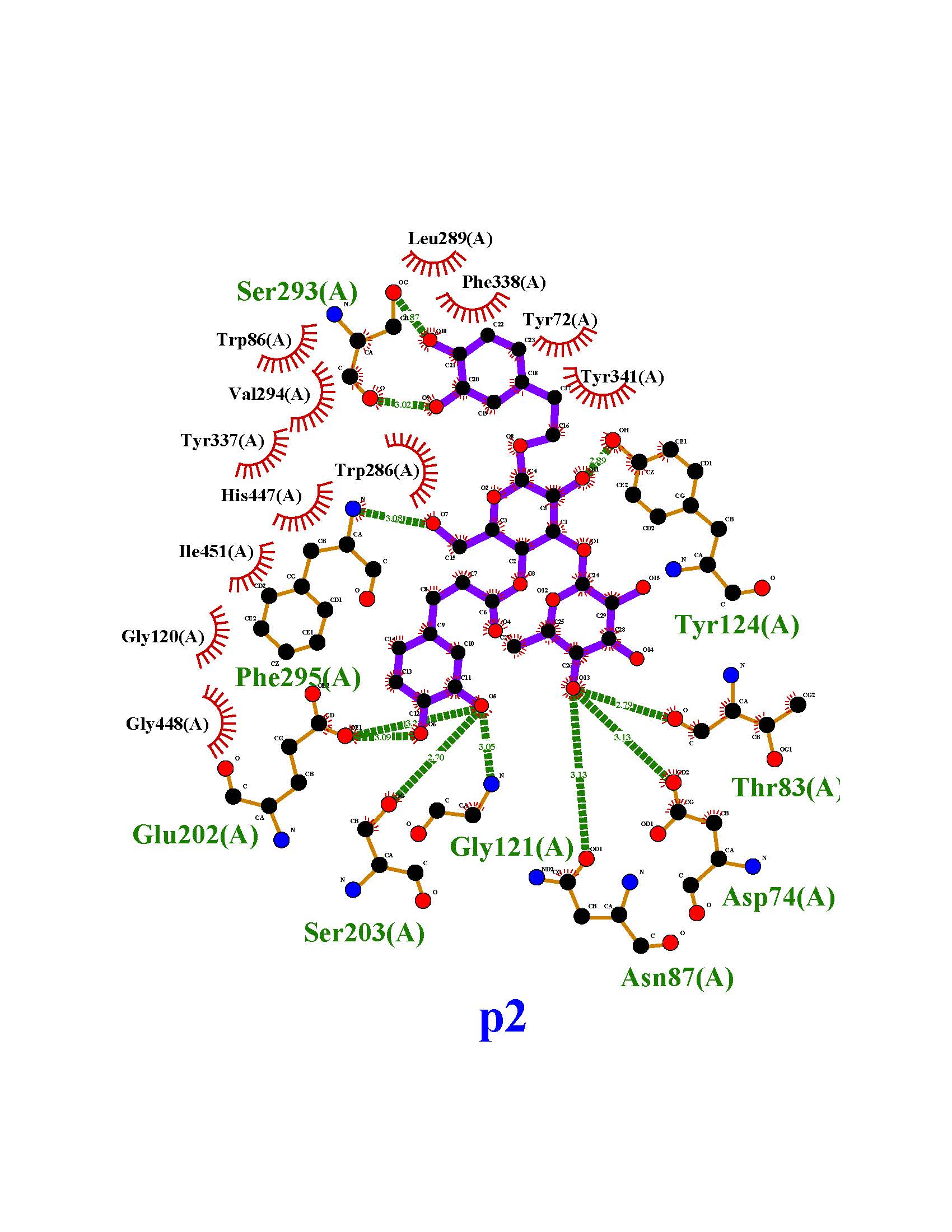

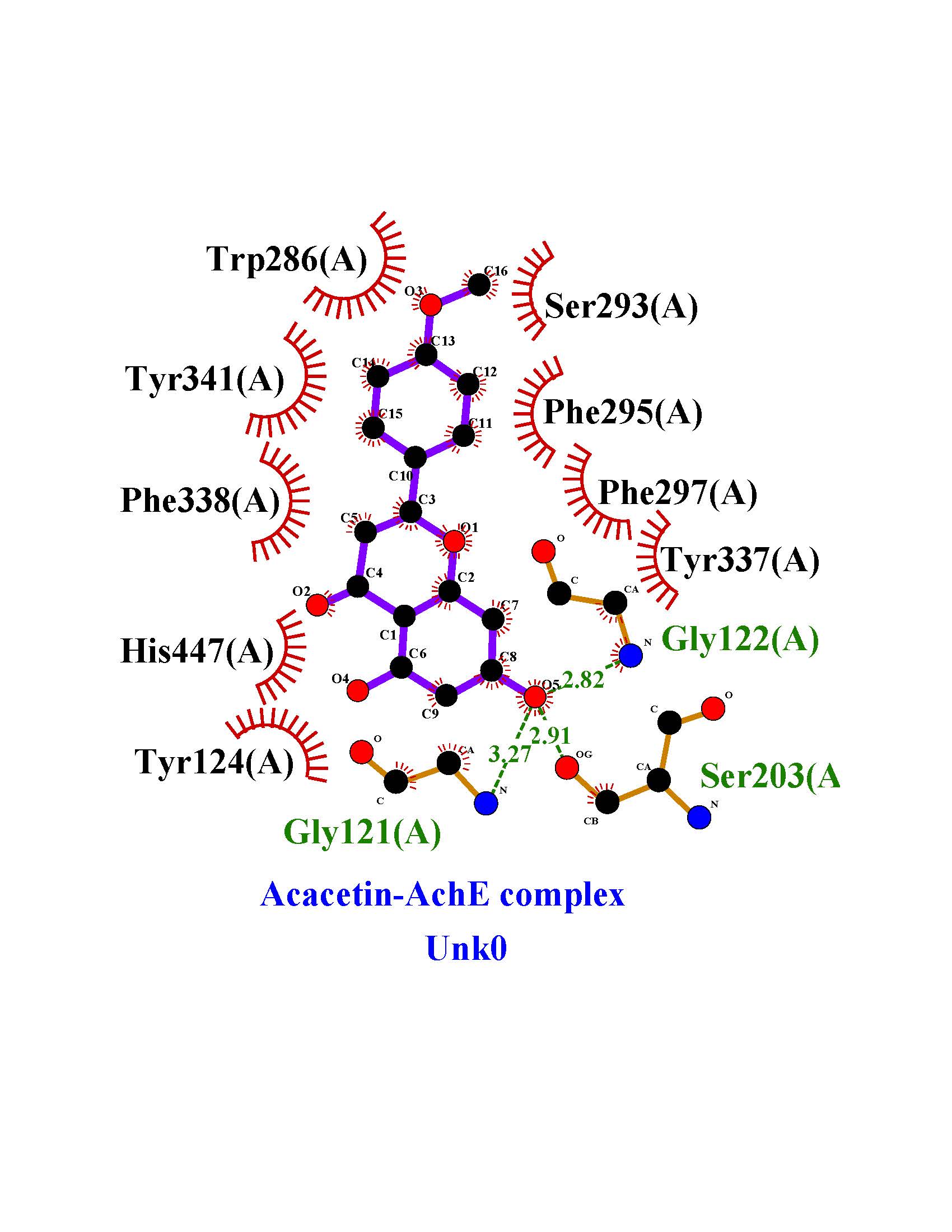

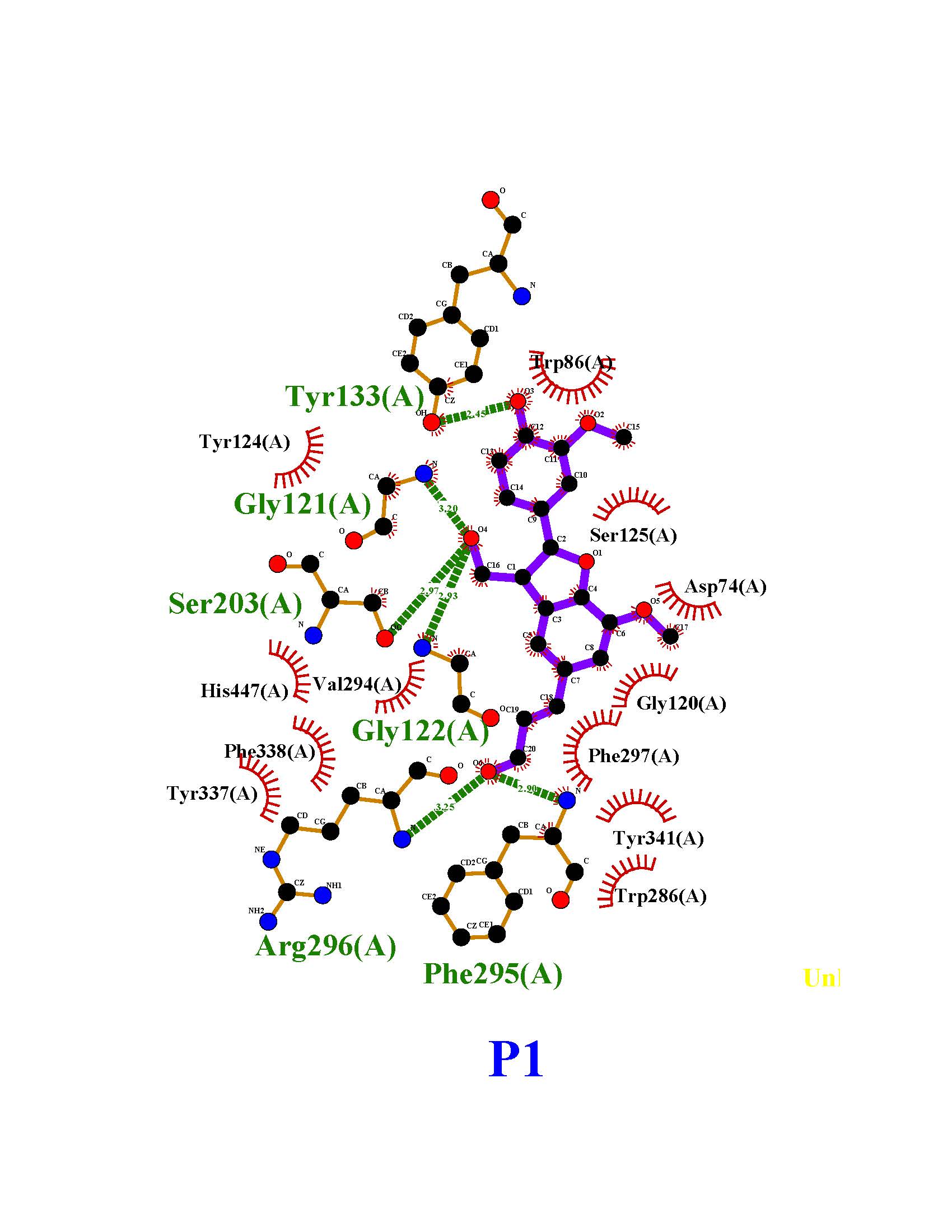


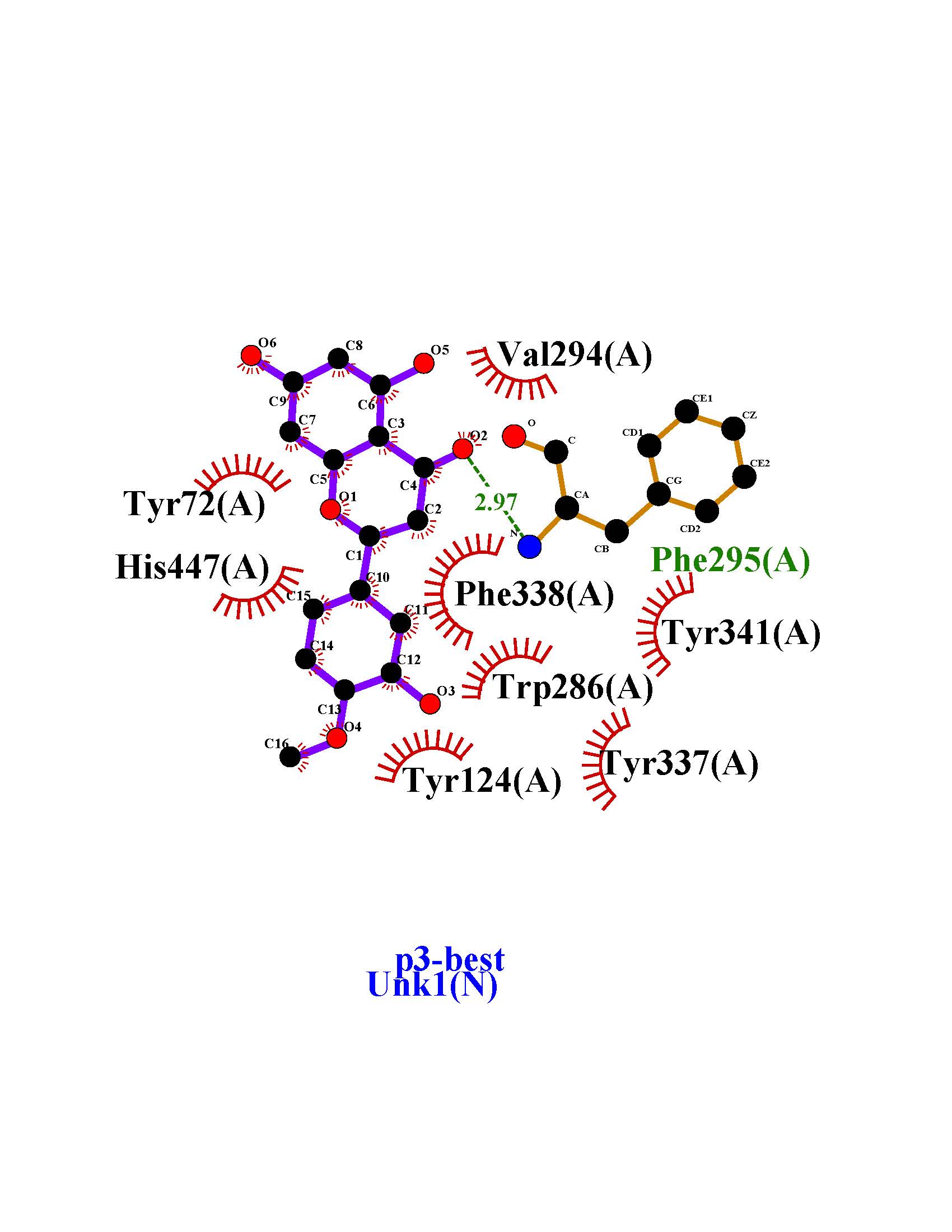

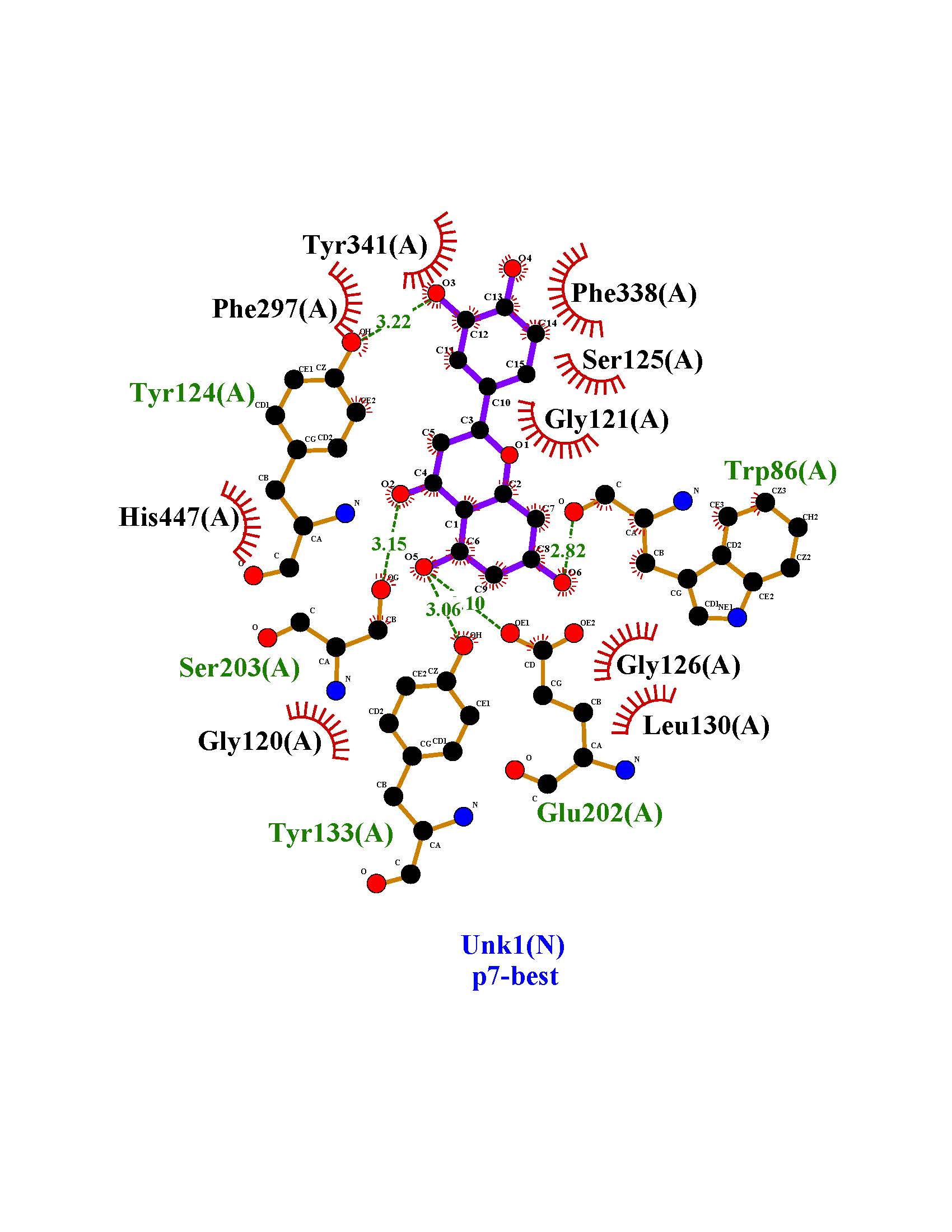

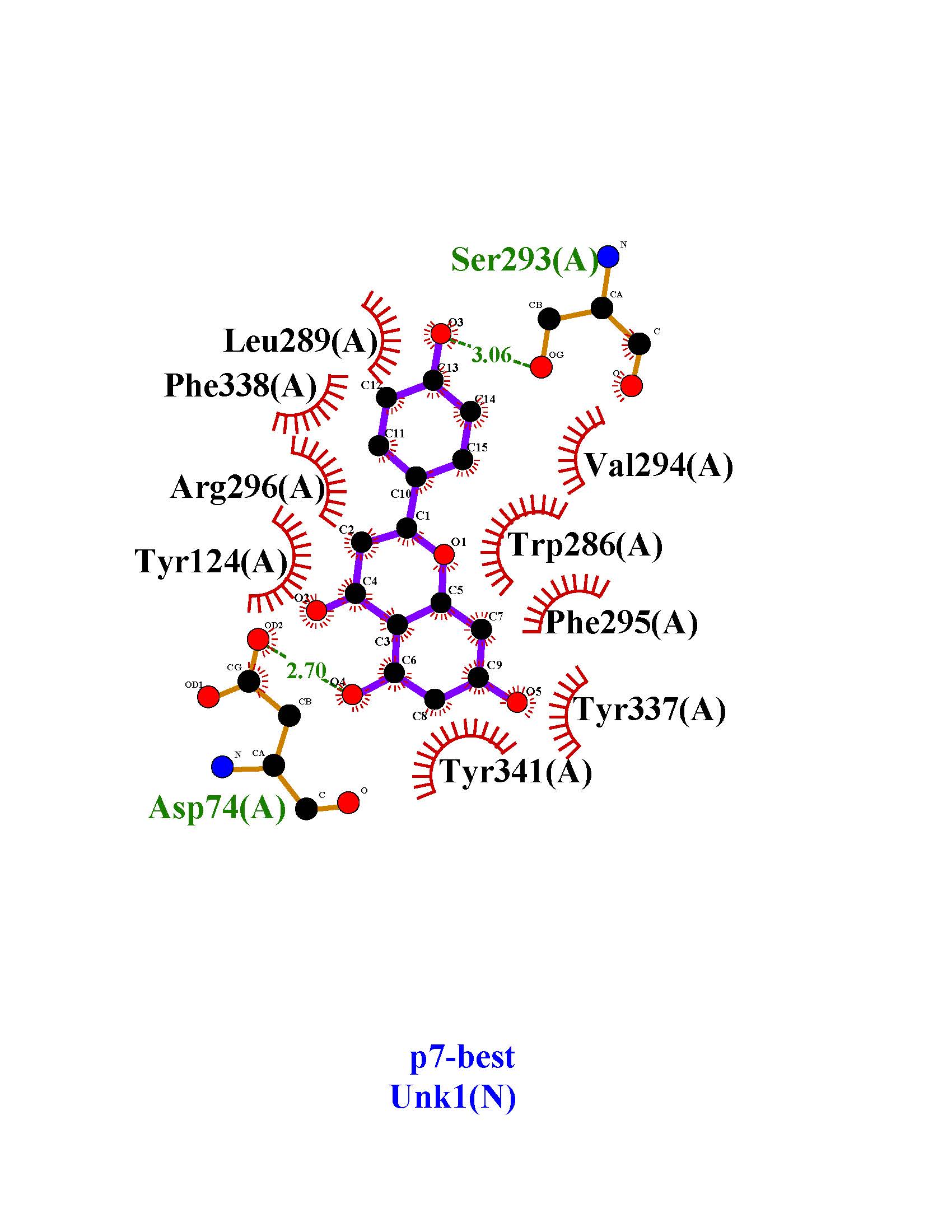


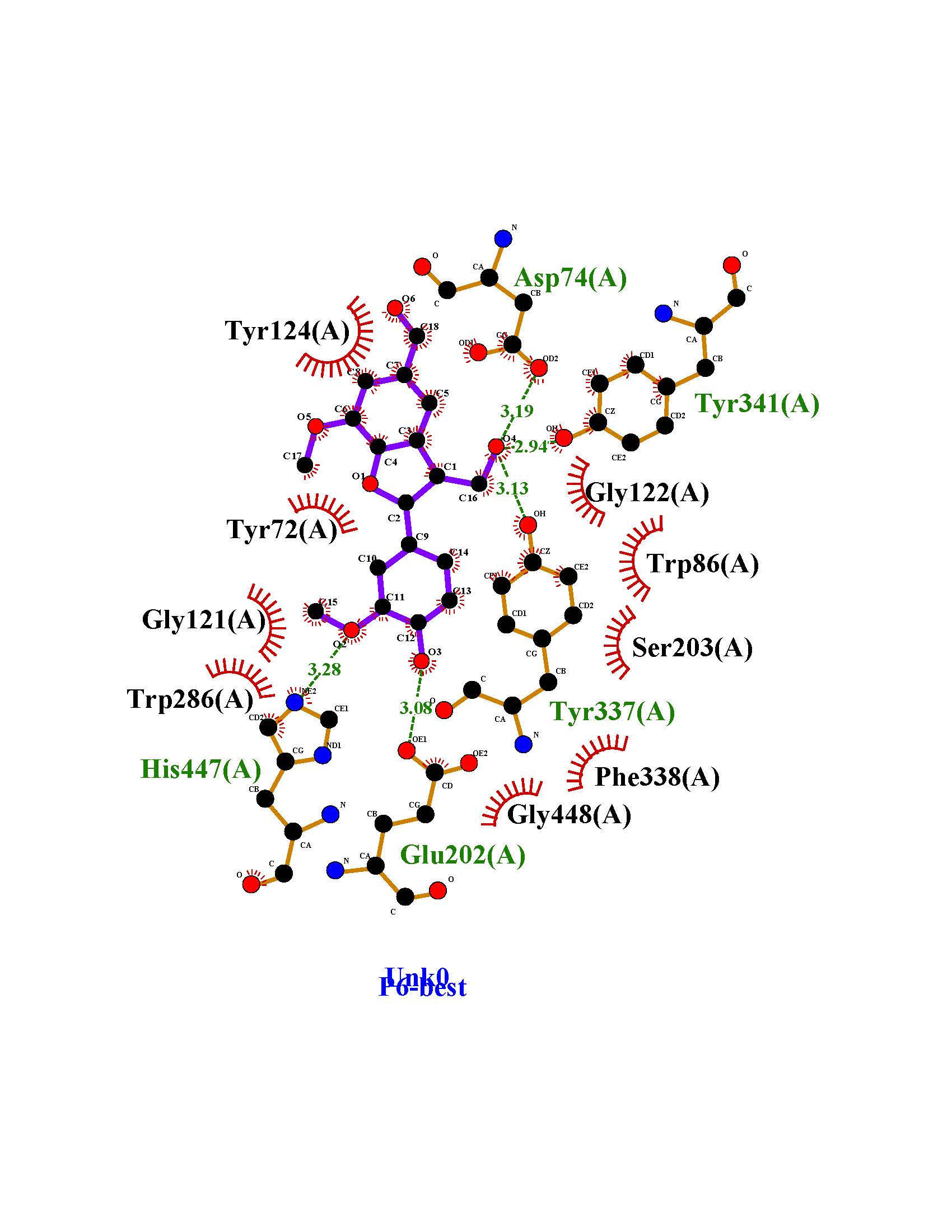

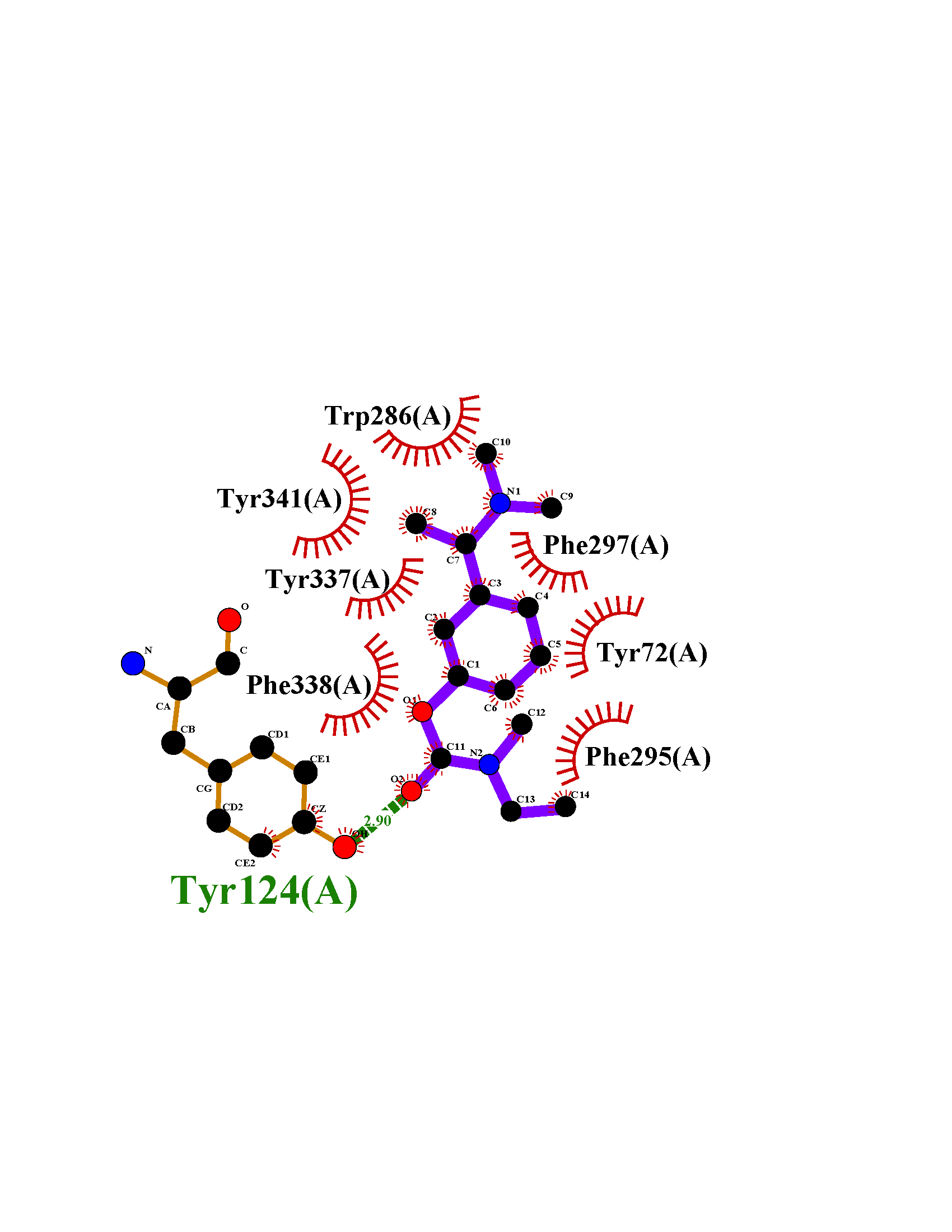


**Supplementary Figure 3:** 2d conformations of acetylcholinesterase enzyme (AChE) with five potential ligands: (A) acteoside, (B) acacetin, (C) balanophonin, (D) hesperetin, (E) luteolin, (F) naringenin, (G) ficusal, (H) rivastigmine (standard) produced by Ligplot+ analysis.

**(A)**

**(B)**

**(C)**

**(D)**


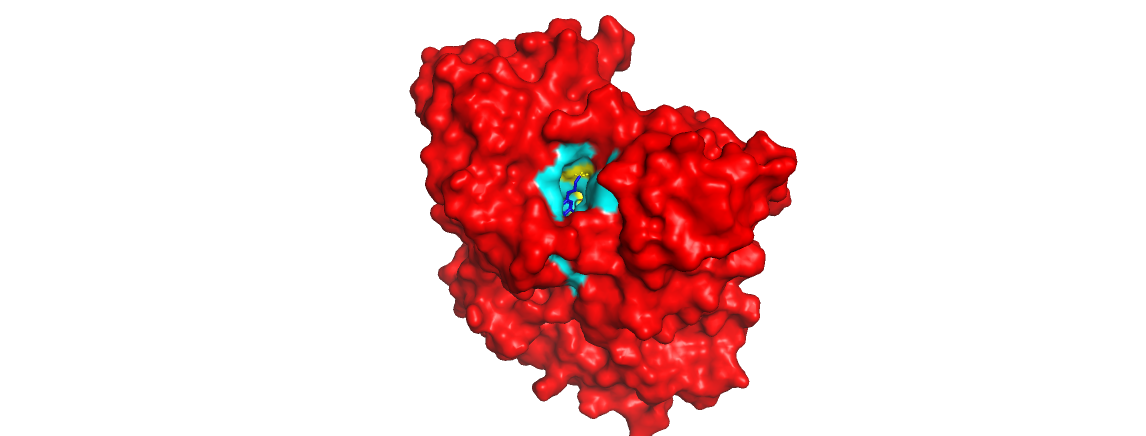

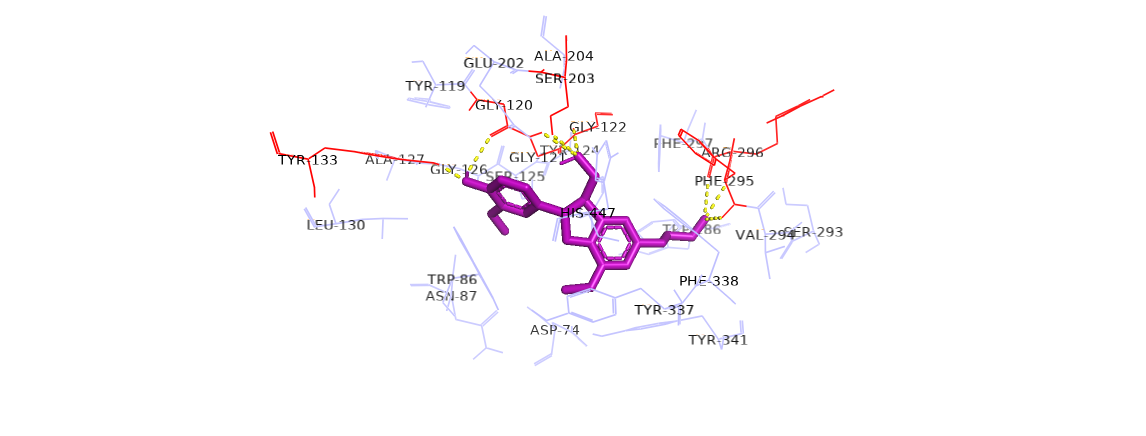


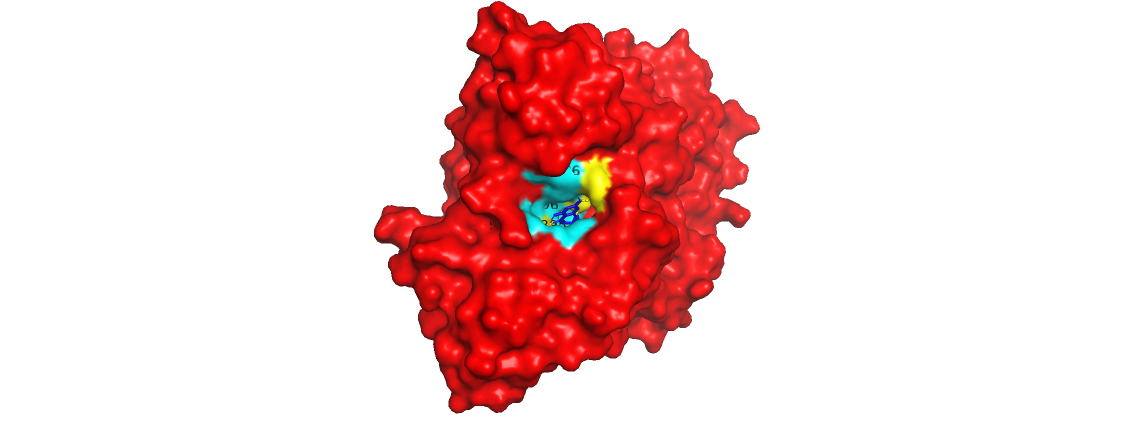

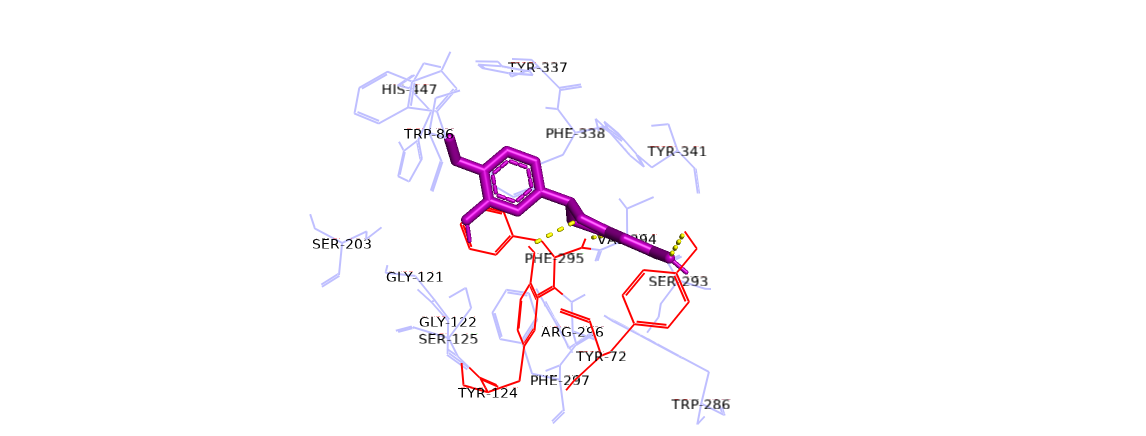


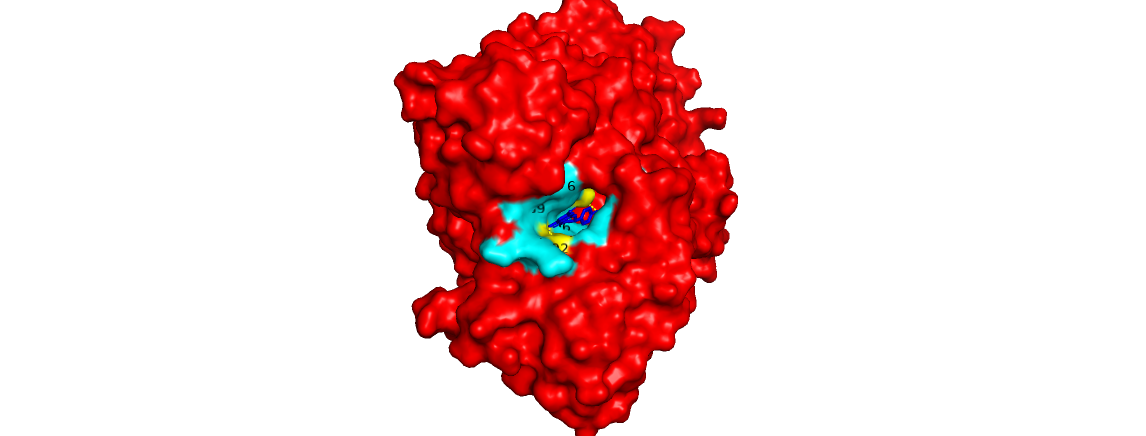

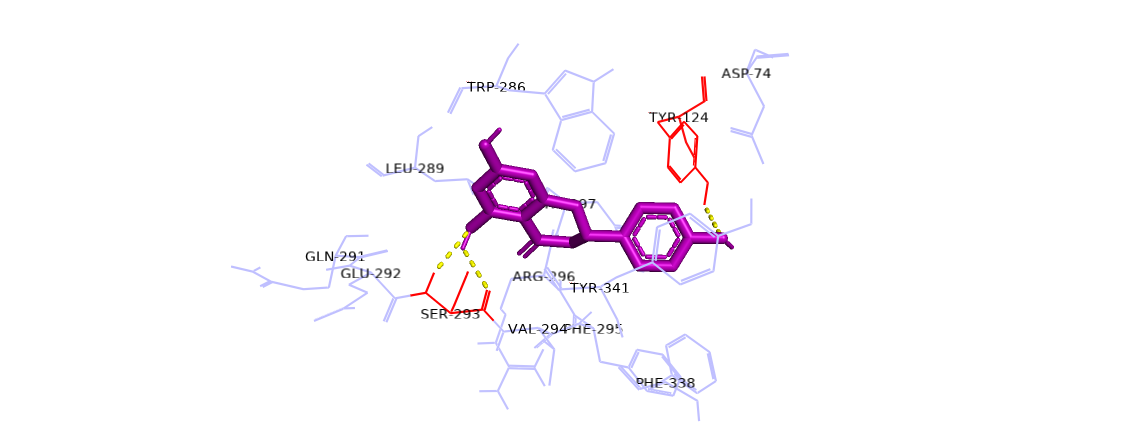


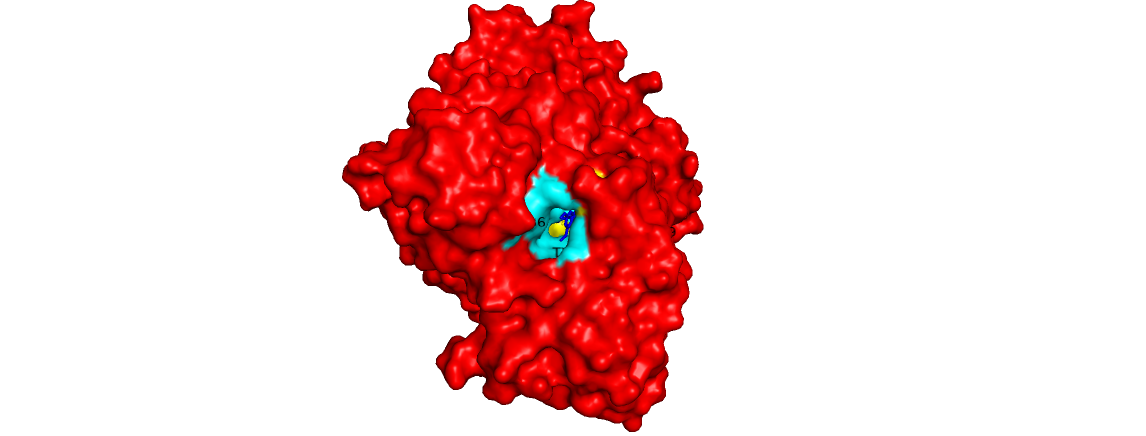

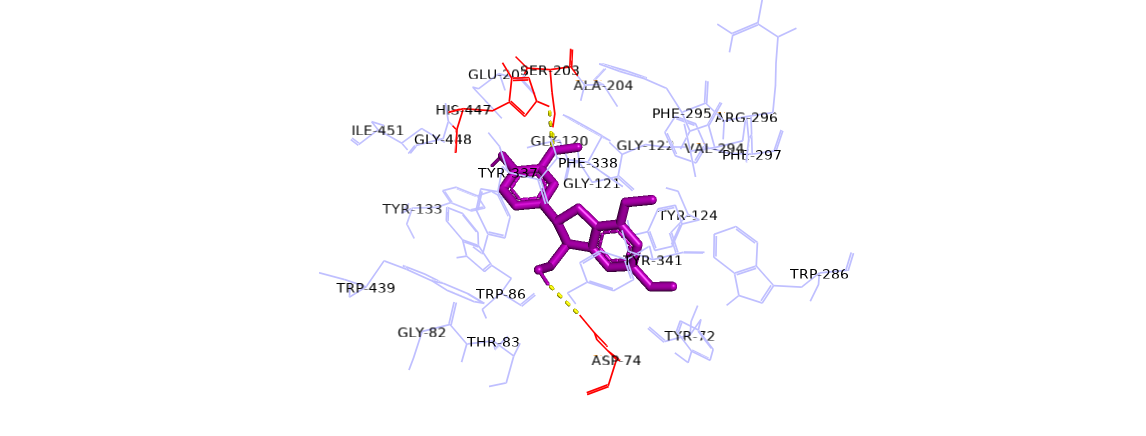


**Supplementary Figure 4:** Possible 3d interactions of (A) balanophonin, (B) hesperetin, (C) naringenin, (D) ficusal with acetylcholinesterase (AchE) (pdb id: 4ey7) (pose predicted by PyMoL).

**Supplementary Figure 5:** Bioavailability radar of lead compounds of *aeginetia indica* with standard rivastigmine as following:(A) acteoside, (B) acacetin, (C) balanophonin, (D) hesperetin, (E) luteolin, (F) naringenin, (G) ficusal, (H) rivastigmine

**(G)**

**(H)**

**(E)**

**(F)**

**(C)**

**(D)**

**(A)**

**(B)**


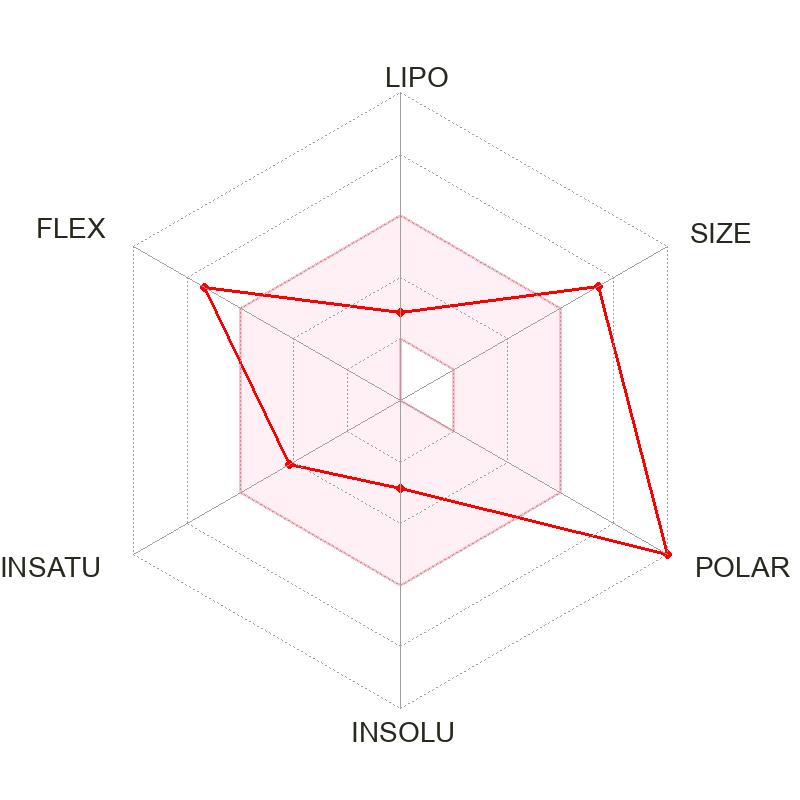

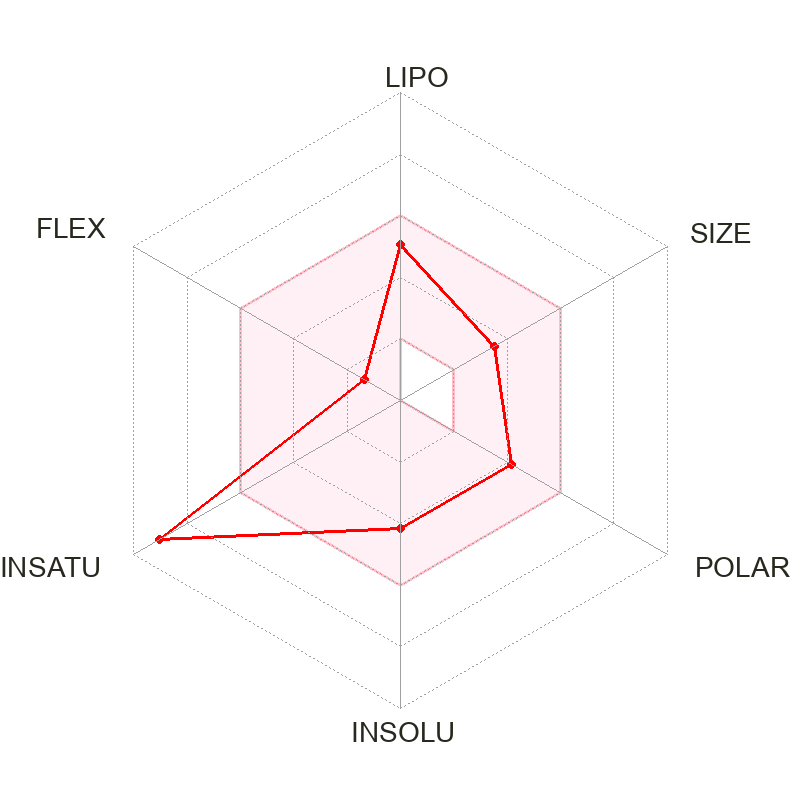


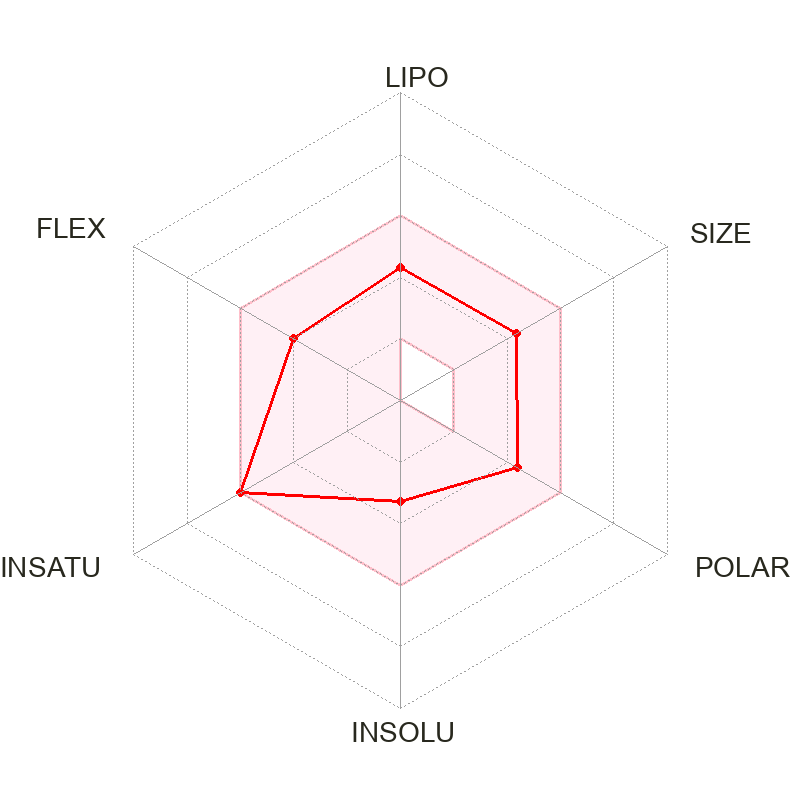

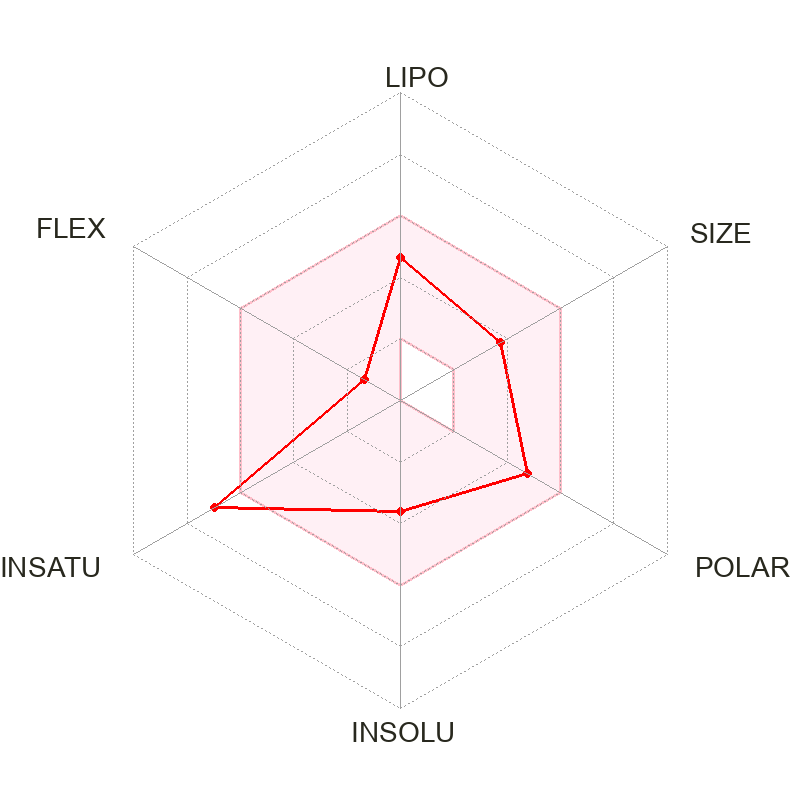


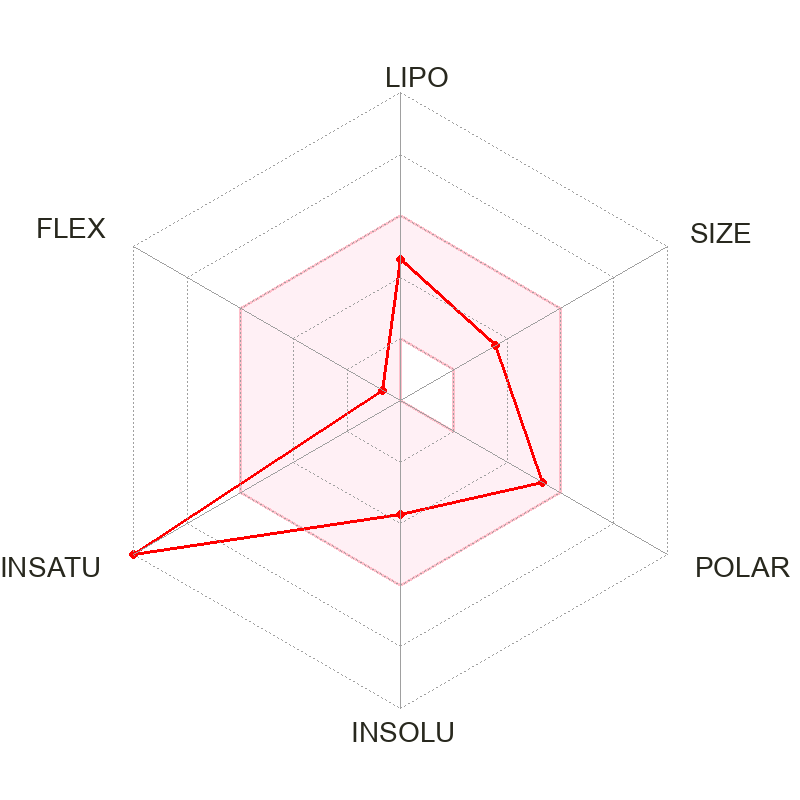

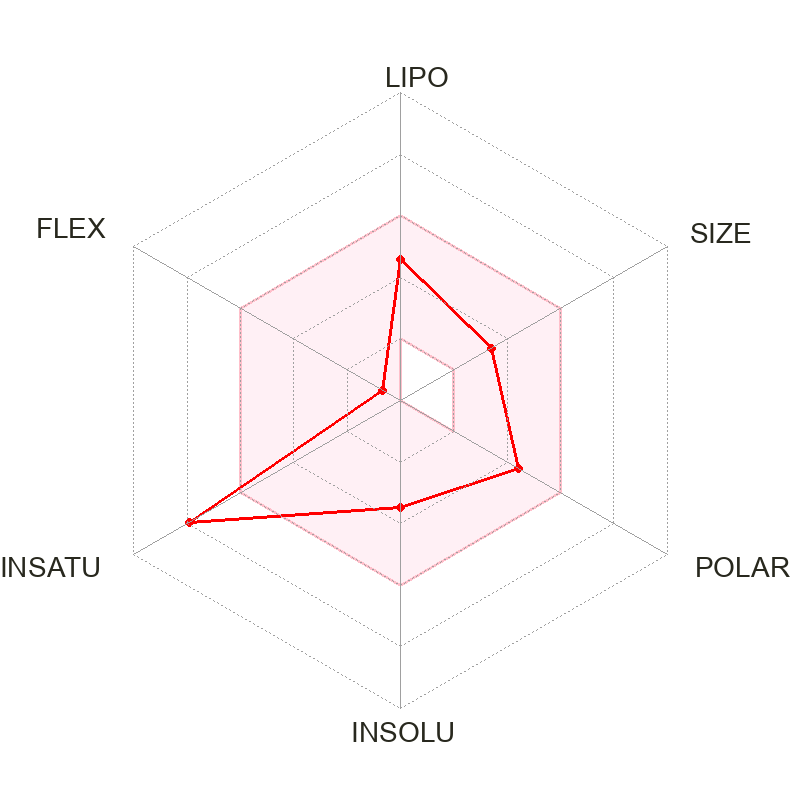


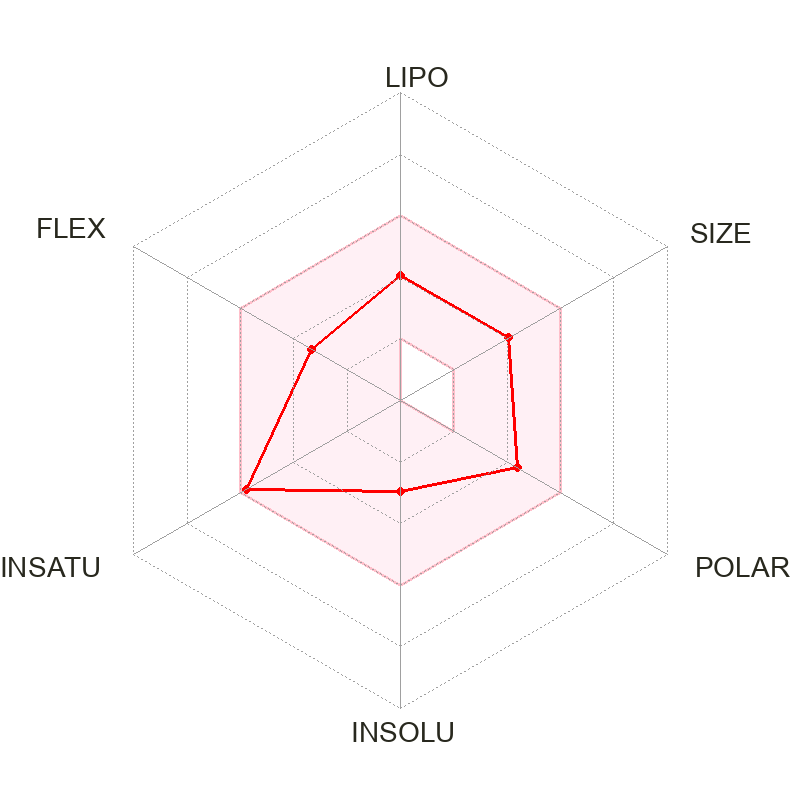

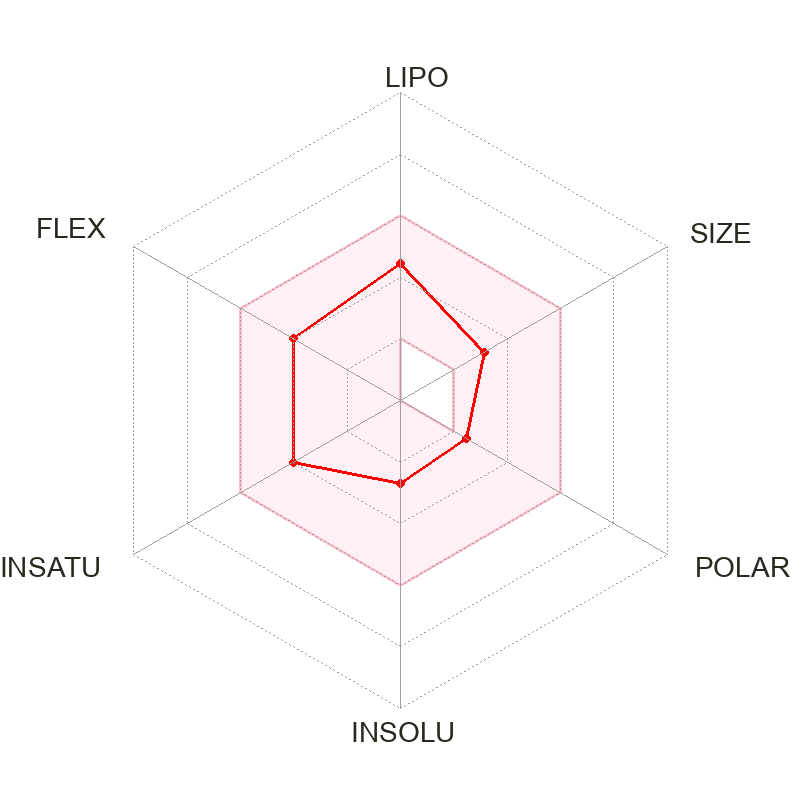


**Supplementary Table 1:** Analytical method validation parameters that belong to the LC-MS/MS method

| No | Analytes | RT*^a^* | M.I. (m/z)*^b^* | F.I. (m/z)*^c^* | Ion  mode | Equation | *r^2d^* | *RSD*%*^e^* | | Linearity Range (mg/L) | *LOD*/*LOQ* (µg/L)*^f^* | Recovery (%) | | *U^g^* | Gr. No*^i^* |
| --- | --- | --- | --- | --- | --- | --- | --- | --- | --- | --- | --- | --- | --- | --- | --- |
|  |  |  |  |  |  |  |  | Inter  day | Intra  day |  |  | Inter  day | Intraday |  |  |
| 1 | Quinic acid | 3.0 | 190.8 | 93.0 | Neg | *y*=-0.0129989+2.97989*×* | 0.996 | 0.69 | 0.51 | 0.1-5 | 25.7/33.3 | 1.0011 | 1.0083 | 0.0372 | 1 |
| 2 | Fumaric aid | 3.9 | 115.2 | 40.9 | Neg | *y*=-0.0817862+1.03467*×* | 0.995 | 1.05 | 1.02 | 1-50 | 135.7/167.9 | 0.9963 | 1.0016 | 0.0091 | 1 |
| 3 | Aconitic acid | 4.0 | 172.8 | 129.0 | Neg | *y*=-0.7014530+32.9994*×* | 0.971 | 2.07 | 0.93 | 0.1-5 | 16.4/31.4 | 0.9968 | 1.0068 | 0.0247 | 1 |
| 4 | Gallic acid | 4.4 | 168.8 | 79.0 | Neg | *y*=0.0547697+20.8152*×* | 0.999 | 1.60 | 0.81 | 0.1-5 | 13.2/17.0 | 1.0010 | 0.9947 | 0.0112 | 1 |
| 5 | Epigallocatechin | 6.7 | 304.8 | 219.0 | Neg | *y*=-0.00494986+0.0483704*×* | 0.998 | 1.22 | 0.73 | 1-50 | 237.5/265.9 | 0.9969 | 1.0040 | 0.0184 | 3 |
| 6 | Protocatechuic acid | 6.8 | 152.8 | 108.0 | Neg | *y*=0.211373+12.8622*×* | 0.957 | 1.43 | 0.76 | 0.1-5 | 21.9/38.6 | 0.9972 | 1.0055 | 0.0350 | 1 |
| 7 | Catechin | 7.4 | 288.8 | 203.1 | Neg | *y*=-0.00370053+0.431369*×* | 0.999 | 2.14 | 1.08 | 0.2-10 | 55.0/78.0 | 1.0024 | 1.0045 | 0.0221 | 3 |
| 8 | Gentisic acid | 8.3 | 152.8 | 109.0 | Neg | *y*=-0.0238983+12.1494*×* | 0.997 | 1.81 | 1.22 | 0.1-5 | 18.5/28.2 | 0.9963 | 1.0077 | 0.0167 | 1 |
| 9 | Chlorogenic acid | 8.4 | 353.0 | 85.0 | Neg | *y*=0.289983+36.3926*×* | 0.995 | 2.15 | 1.52 | 0.1-5 | 13.1/17.6 | 1.0000 | 1.0023 | 0.0213 | 1 |
| 10 | Protocatechuic aldehyde | 8.5 | 137.2 | 92.0 | Neg | *y*=0.257085+25.4657*×* | 0.996 | 2.08 | 0.57 | 0.1-5 | 15.4/22.2 | 1.0002 | 0.9988 | 0.0396 | 1 |
| 11 | Tannic acid | 9.2 | 182.8 | 78.0 | Neg | *y*=0.0126307+26.9263*×* | 0.999 | 2.40 | 1.16 | 0.05-2.5 | 15.3/22.7 | 0.9970 | 0.9950 | 0.0190 | 1 |
| 12 | Epigallocatechin gallate | 9.4 | 457.0 | 305.1 | Neg | *y*=-0.0380744+1.61233*×* | 0.999 | 1.30 | 0.63 | 0.2-10 | 61.0/86.0 | 0.9981 | 1.0079 | 0.0147 | 3 |
| 13 | 1,5-dicaffeoylquinic acid | 9.8 | 515.0 | 191.0 | Neg | *y*=-0.0164044+16.6535*×* | 0.999 | 2.42 | 1.48 | 0.1-5 | 5.8/9.4 | 0.9983 | 0.9997 | 0.0306 | 1 |
| 14 | 4-OH Benzoic acid | 10.5 | 137,2 | 65.0 | Neg | *y*=-0.0240747+5.06492*×* | 0.999 | 1.24 | 0.97 | 0.2-10 | 68.4/88.1 | 1.0032 | 1.0068 | 0.0237 | 1 |
| 15 | Epicatechin | 11.6 | 289.0 | 203.0 | Neg | *y*=-0.0172078+0.0833424*×* | 0.996 | 1.47 | 0.62 | 1-50 | 139.6/161.6 | 1.0013 | 1.0012 | 0.0221 | 3 |
| 16 | Vanilic acid | 11.8 | 166.8 | 108.0 | Neg | *y*=-0.0480183+0.779564*×* | 0.999 | 1.92 | 0.76 | 1-50 | 141.9/164.9 | 1.0022 | 0.9998 | 0.0145 | 1 |
| 17 | Caffeic acid | 12.1 | 179.0 | 134.0 | Neg | *y*=0.120319+95.4610*×* | 0.999 | 1.11 | 1.25 | 0.05-2.5 | 7.7/9.5 | 1.0015 | 1.0042 | 0.0152 | 1 |
| 18 | Syringic acid | 12.6 | 196.8 | 166.9 | Neg | *y*=-0.0458599+0.663948*×* | 0.998 | 1.18 | 1.09 | 1-50 | 82.3/104.5 | 1.0006 | 1.0072 | 0.0129 | 1 |
| 19 | Vanillin | 13.9 | 153.1 | 125.0 | Poz | *y*=0.00185898+20.7382*×* | 0.996 | 1.10 | 0.85 | 0.1-5 | 24.5/30.4 | 1.0009 | 0.9967 | 0.0122 | 1 |
| 20 | Syringic aldehyde | 14.6 | 181.0 | 151.1 | Neg | *y*=-0.0128684+7.90153*×* | 0.999 | 2.51 | 0.77 | 0.4-20 | 19.7/28.0 | 1.0001 | 0.9964 | 0.0215 | 1 |
| 21 | Daidzin | 15.2 | 417.1 | 199.0 | Poz | *y*=9.45747+152.338*×* | 0.996 | 2.25 | 1.32 | 0.05-2.5 | 7.0/9.5 | 0.9955 | 1.0017 | 0.0202 | 2 |
| 22 | Epicatechin gallate | 15.5 | 441.0 | 289.0 | Neg | *y*=-0.0142216+1.06768*×* | 0.997 | 1.63 | 1.28 | 0.1-5 | 19.5/28.5 | 0.9984 | 0.9946 | 0.0229 | 3 |
| 23 | Piceid | 17.2 | 391.0 | 135/106.9 | Poz | *y*=0.00772525+25.4181*×* | 0.999 | 1.94 | 1.16 | 0.05-2.5 | 13.8/17.8 | 1.0042 | 0.9979 | 0.0199 | 1 |
| 24 | *p*-Coumaric acid | 17.8 | 163.0 | 93.0 | Neg | *y*=0.0249034+18.5180*×* | 0.999 | 1.92 | 1.43 | 0.1-5 | 25.9/34.9 | 1.0049 | 1.0001 | 0.0194 | 1 |
| 25 | Ferulic acid-D3-IS*^h^* | 18.8 | 196.2 | 152.1 | Neg | N.A. | N.A. | N.A. | N.A. | N.A. | N.A. | N.A. | N.A. | 0.0170 | 1 |
| 26 | Ferulic acid | 18.8 | 192.8 | 149.0 | Neg | *y*=-0.0735254+1.34476*×* | 0.999 | 1.44 | 0.53 | 1-50 | 11.8/15.6 | 0.9951 | 0.9976 | 0.0181 | 1 |
| 27 | Sinapic acid | 18.9 | 222.8 | 193.0 | Neg | *y*=-0.0929932+0.836324*×* | 0.999 | 1.45 | 0.52 | 0.2-10 | 65.2/82.3 | 1.0031 | 1.0037 | 0.0317 | 1 |
| 28 | Coumarin | 20.9 | 146.9 | 103.1 | Poz | *y*=0.0633397+136.508*×* | 0.999 | 2.11 | 1.54 | 0.05-2.5 | 214.2/247.3 | 0.9950 | 0.9958 | 0.0383 | 1 |

*^a^*R.T.: Retention time, *^b^*MI (*m/z):* Molecular ions of the standard analytes (m/z ratio), *^c^*FI (*m/z):* Fragment ions *^d^r^2^*: Coefficient of determination, *^e^RSD*: Relative standard deviation, *^f^LOD*/*LOQ* (µg/L): Limit of detection/quantification, *^g^U* (%): percent relative uncertainty at 95% confidence level (*k* = 2), *^h^*IS: Internal standard, *^i^*Gr. No: Represents grouping of internal standards, these numbers indicate which IS stands for which phenolic compound.

**Supplementary Table 2:** Lists of previously identified components from *Aeginetia indica* for their different bioactivities.

| SL | **Target Ligands** | **Pubchem ID** | **References** |
| --- | --- | --- | --- |
| 1 | Aeginetic acid | 15693867 | - Dighe, S. S.; Kulkarni, A. B. IndianJ.Chem. 1973, 11,404. - Dighe, S. S.; Kulkarni, A. B. IndianJ.Chem. 1974, 12,413. - Dighe, S. S.; Kulkarni, A. B. IndianJ.Chem. 1974, 12,414. - Dighe, S. S.; Manerikar, S. V.; Kulkarni, A. B. Indian J. Chem., Sect. B. 1977, 15B, 546. - Dighe, S. S.; Manerikar, S. V.; Kulkarni, A. B. Indian J. Chem., Sect. B. 1977, 15B, 550. |
| 2 | Aeginetolide | 15948056 |  |
| 3 | Polyene D | Not found |  |
| 4 | Polyene F | Not found |  |
| 5 | Polyene E | Not found | - Dighe, S. S.; Manerikar, S. V.; Kulkarni, A. B. Indian J. Chem., Sect. B. 1977, 15B, 550. - Y. OsHIMA, Y. KAwAKAMI, Y. KIso, Hir. HIKINo, L.-L. YANG, K.-Y. YEN, Antihepatotoxic Principles of Aeginetia indica Herbs, Japanese Soc. Pharmacogn. 38 (1984) 198–200. |
| 6 | Hydroxy- β –ionone glucoside | Not found | - Endo, Thoru & Taguchi, Heihachiro & Sasaki, Hiroshi & Yosioka, Itiro. (1979). Studies on the Constituents of Aeginetia indica L. var. gracilis Nakai. Structures of Three Glycosides isolated from the Whole Plant. CHEMICAL & PHARMACEUTICAL BULLETIN. 27. 2807-2814. 10.1248/cpb.27.2807. |
| 7 | Aeginetoside | Not found |  |
| 8 | Isoaucubin | Not found |  |
| 9 | β-Sitosterol | 222284 | - Y. OsHIMA, Y. KAwAKAMI, Y. KIso, Hir. HIKINo, L.-L. YANG, K.-Y. YEN, Antihepatotoxic Principles of Aeginetia indica Herbs, Japanese Soc. Pharmacogn. 38 (1984) 198–200. |
| 11 | Apigenin | 5280443 |  |
| 12 | β -sitosteryl glucoside | 5742590 |  |
| 13 | Balanophonin 4-O- β -D-glucopyranoside | Not found | - Ho, Jiau‐Ching & Chen, Chiu‐Ming & Row, Lie‐Ching. (2003). Neolignans from the Parasitic Plants. Part 1. Aeginetia Indica. Journal of the Chinese Chemical Society. 50. 10.1002/jccs.200300183. |
| 14 | Aegineoside | 132353271 |  |
| 15 | Dehydrodiconiferyl alcohol 4-O- β-D-glucopyranoside | Not found |  |
| 16 | Dehydrodiconiferyl alcohol γ'-O- β-D-glucopyranoside | Not found |  |
| 17 | Balanophonin | 23252258 |  |
| 18 | Ficusal | 10496641 |  |
| 19 | α-L-rhamnopyranosyl-(1→3)-1-O-caffeoyl- β-D-glucopyranoside | Not found | - Ho, Jiau‐Ching & Chen, Chiu‐Ming & Li, Zhi‐Qiang & Row, Lie‐Ching. (2004). Phenylpropanoid Glycosides from the Parasitic Plant, Aeginetia Indica. Journal of the Chinese Chemical Society. 51. 10.1002/jccs.200400160. |
| 20 | 2",3"'-diacetyl acteoside | Not found |  |
| 21 | 2´-acetyl acteoside | Not found |  |
| 22 | Cistanoside C | Not found |  |
| 23 | Acteoside | 5281800 |  |
| 24 | Trans-coni-feraldehyde | 5280536 |  |
| 25 | Vanillic acid | 8468 |  |
| 26 | P-hydroxybenzadehyde | Not found |  |
| 27 | (5R,6R)-5,6-dihydroxy-5,6-dihydro- β -ionone | Not found |  |

**Supplementary Table 3:** Bond analysis of the selected phytoconstituents and rivastigmine (Standard) with acetylcholinesterase Enzyme (AChE)

| Compound | Structure | Binding Affinity | Types of Interactions | | | |
| --- | --- | --- | --- | --- | --- | --- |
| Hesperetin (CID: 72281) | 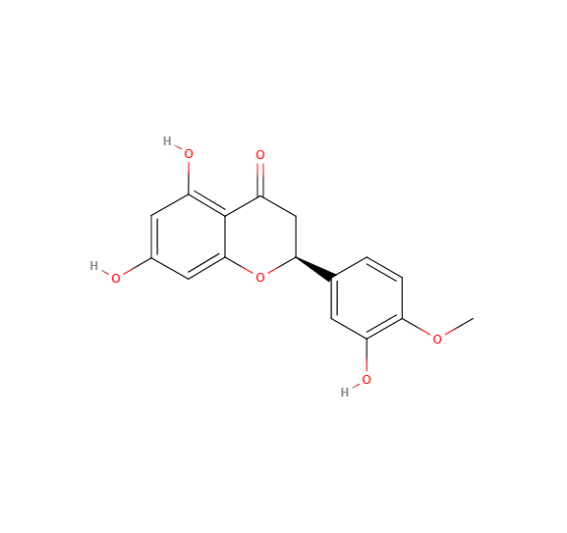 | -10.4 | **H Bonding Residue**  **(Distance)** | | **Total H Bond** | **Hydrophobic Bond Residue (Type)** |
|  |  |  | **Conventional** | Tyr72 (2.77)  Tyr124 (2.82)  Phe295 (2.07) | 5 | TRP286  TRP286  PHE338  TYR341  TYR337  TYR341 |
|  |  |  | **carbon** | Val294 (2.50)  His447 (2.65) |  |  |
| Hesperidin (CID: 10621) | 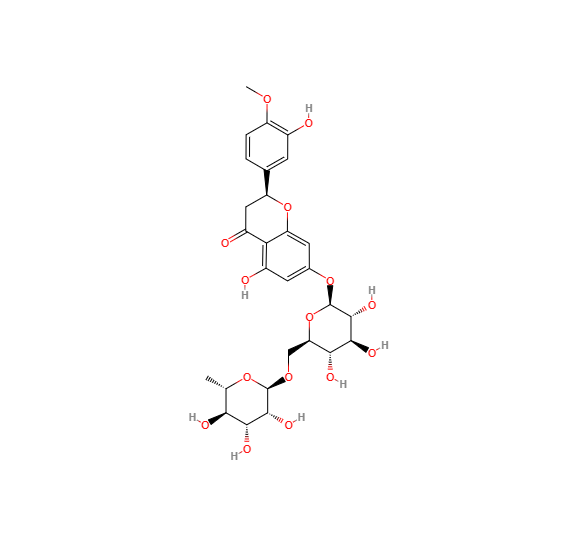 | -10.9 | **Conventional** | Tyr72 (2.86)  Phe295 (2.08)  Ser293 (1.94) | 8 | Trp286  Trp286  Tyr341  Trp86  Tyr124  Leu289  Trp286  Trp286 |
|  |  |  | **Carbon** | Glu292 (2.89)  Tyr341 (2.41)  Tyr341 (2.61)  Ser293 (3.02)  Tyr341 (2.41) |  |  |
| Naringenin (CID: 439246) | 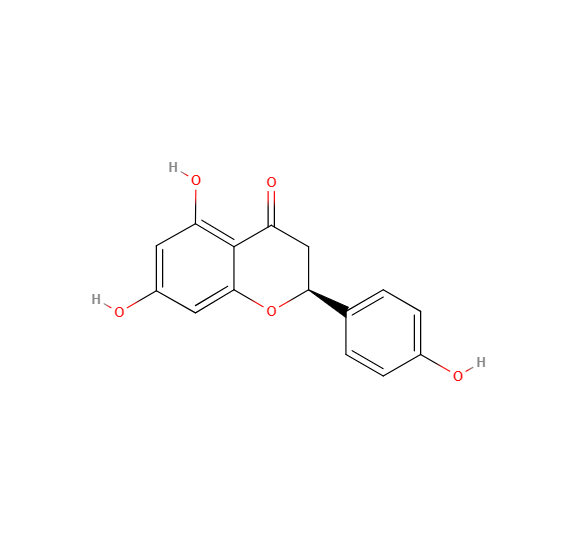 | -10.3 | **Conventional** | Tyr341 (2.94)  Tyr124 (2.87) | 3 | Trp286  Tyr341 |
|  |  |  | **Carbon** | Phe338 (2.60) |  |  |
| Aegineoside (CID:132353271) | 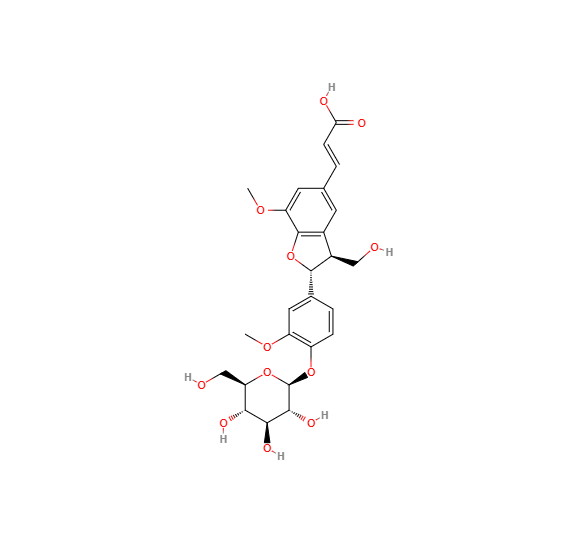 | -10.7 | **Conventional** | Tyr72 (2.1758)  Thr75 (3.03441)  Gly122 (2.25375)  Phe295 (2.11832)  His447 (2.40334)  Asp74 (2.40995) | 11 | Tyr124  Trp286 Trp286  Tyr341  Tyr124  Phe338  Tyr341 |
|  |  |  | **Carbon** | Ser203 (2.29539)  Val294 (3.05877)  Tyr72 (3.00817)  Tyr341 (2.50204)  Asp74 (3.06745) |  |  |
| Aeginetic Acid (CID:15693867) | 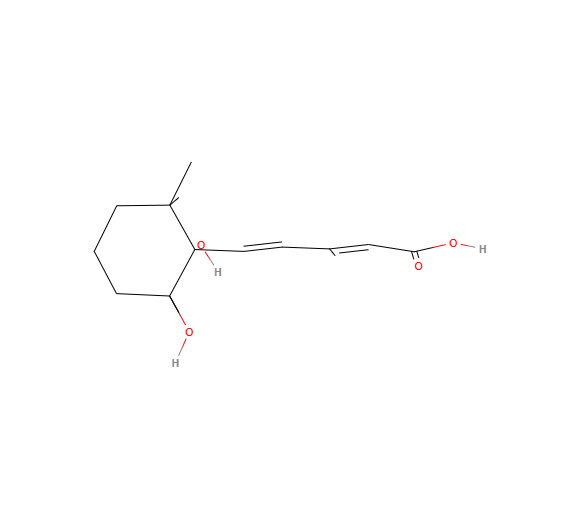 | -8.7 | **Conventional** | Ser293 (2.30159)  Arg296 (2.66882)  Ser293 (2.08605)  Arg296 (2.26433) | 5 | Leu289  Leu289  Trp286  Trp286  Trp286  Tyr341 |
|  |  |  | **Carbon** | Glu292 (2.92129) |  |  |
| Aeginetolide (CID:15948056) | 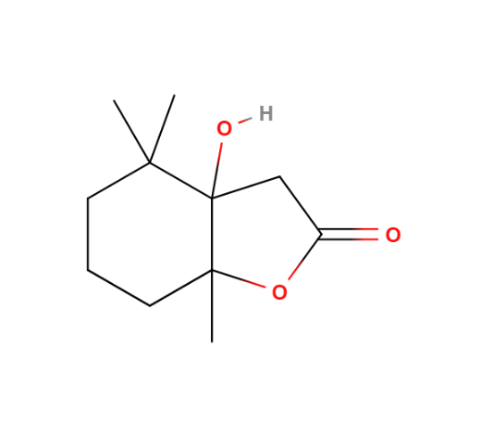 | -8.5 | **Conventional** | Gly121 (2.54648)  Gly122 (2.46628)  Tyr124 (2.33683)  Ser203 (2.18058) | 4 | Trp86  Trp86  Tyr124  Tyr337  Phe338  Phe338  Tyr341  His441 |
| Apigenin (CID:5280443) | 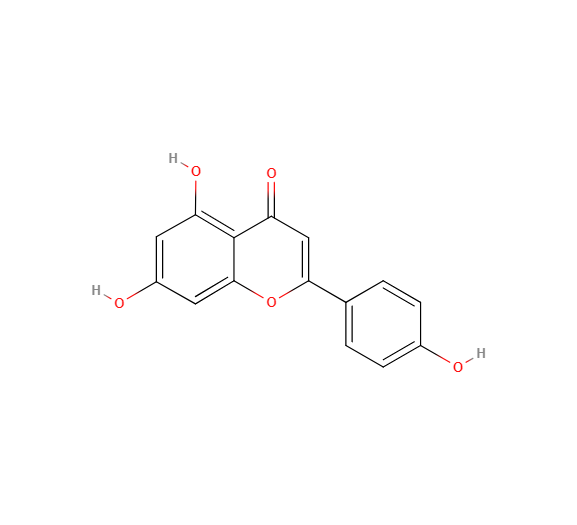 | -10.2 | **Conventional** | Phe295 (2.50924)  Tyr341 (2.86385) | 5 | Trp286  Trp286  Trp286  Phe338  Tyr341  Tyr341  Tyr337  Tyr341 |
|  |  |  | **Carbon** | Val294 (2.10425)  His447 (2.66533)  Tyr124 (3.1372) |  |  |
| Balanophonin (CID:23252258) | 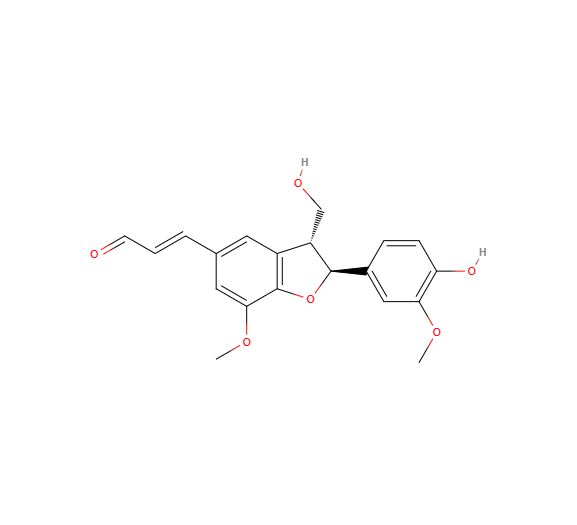 | -10.1 | **Conventional** | Gly122 (2.31638)  Tyr133 (1.77431)  Phe295 (2.36095)  Arg296 (2.31695)  Gly120 (2.49201) | 10 | Tyr337  Phe338  Tyr341 |
|  |  |  | **Carbon** | Val294 (2.95192)  Trp86 (2.47829)  Ser125 (2.19912)  Tyr337 (3.01899)  Asp74 (2.71813) |  |  |
| Ficusal (CID:10496641) | 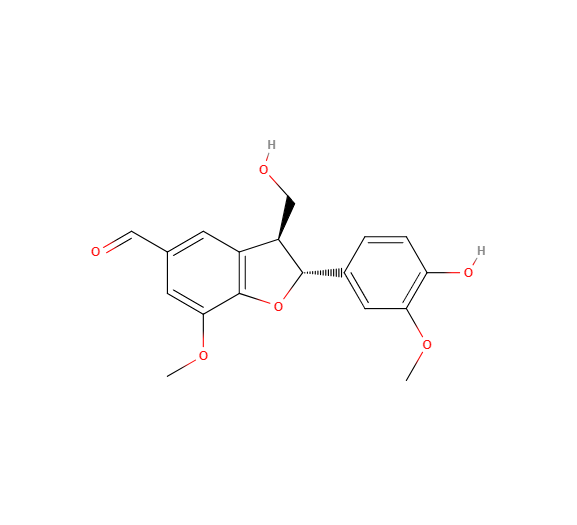 | -9.0 | **Conventional** | Tyr72 (2.52871)  His447 (2.38528)  Tyr124 (2.57267) | 7 | Trp286  Trp286  Tyr341  Tyr124 |
|  |  |  | **Carbon** | His447 (2.24183)  Asp74 (2.62101)  Arg296 (2.81227)  Tyr124 (3.23429) |  |  |
